# Comprehensive Morpho-Functional Profiling of Peruvian Andean *Capsicum pubescens* Germplasm Reveals Promising Accessions with High Agronomic and Nutraceutical Value

**DOI:** 10.3390/plants15020288

**Published:** 2026-01-17

**Authors:** Erick Leao Salas-Zeta, Katherine Lisbeth Bernal-Canales, Andrea Delgado-Lazo, Gonzalo Pacheco-Lizárraga, Marián Hermoza-Gutiérrez, Hector Cántaro-Segura, Elizabeth Fernandez-Huaytalla, Dina L. Gutiérrez-Reynoso, Fredy Quispe-Jacobo, Karina Ccapa-Ramirez

**Affiliations:** 1 Dirección de Recursos Genéticos y Biotecnología, Instituto Nacional de Innovación Agraria (INIA), Lima 15024, Peru; eleaosz@gmail.com (E.L.S.-Z.); l26isbeth@gmail.com (K.L.B.-C.); dr.marian.hermoza@gmail.com (M.H.-G.); hcantarosegura@gmail.com (H.C.-S.); efernandezh@inia.gob.pe (E.F.-H.); dgutierrez@inia.gob.pe (D.L.G.-R.); 2 Estación Experimental Agraria Arequipa, Dirección de Recursos Genéticos y Biotecnología, Instituto Nacional de Innovación Agraria (INIA), Arequipa 04011, Peru; andreadelgadolazo@gmail.com (A.D.-L.); gpacheco@inia.gob.pe (G.P.-L.); 3 Laboratorio de Investigación Nutricional de los Recursos Genéticos, Dirección de Recursos Genéticos y Biotecnología, Instituto Nacional de Innovación Agraria (INIA), Lima 15024, Peru; fredyenrique@gmail.com

**Keywords:** *Capsicum pubescens*, phenotypic diversity, bioactive compounds, carotenoids, capsaicinoids, antioxidants, crop improvement

## Abstract

*Capsicum pubescens* (rocoto) is an Andean domesticate with notable agronomic and nutraceutical potential, yet it remains underrepresented in chili pepper breeding programs. In this study, 78 accessions from the Peruvian Andes were evaluated in a single field environment during the 2024 growing season for 28 variables spanning plant architecture, phenology and yield, color (CIELAB), weight, fruit morphology, physicochemical variables, and functional phytochemicals, including total phenolics, carotenoids, ascorbic acid, capsaicinoids, and antioxidant activity (FRAP, DPPH, ABTS). Descriptive analyses revealed broad phenotypic diversity in key variables such as yield and bioactive compounds. Spearman correlations uncovered a clear modular structure, with strong within-domain associations across morphological, chromatic, and biochemical variables, and statistically significant but low-magnitude cross-domain associations (e.g., fruit length with pungency, redness with total phenolics). Principal component analysis and hierarchical clustering resolved three differentiated phenotypic profiles: (i) low-pungency accessions with high soluble solids and varied fruit colors; (ii) highly pungent materials with elevated antioxidant capacity; and (iii) large, red-fruited accessions with considerable carotenoid content and high moisture. This multivariate architecture revealed weak cross-block correlations among agronomic, color, and functional traits, enabling selection of promising accessions combining desirable agronomic attributes and favorable bioactive profiles in specific accessions. These results provide a quantitative foundation for future breeding strategies in *C. pubescens*, opening concrete opportunities to develop improved cultivars that simultaneously meet productivity and functional quality criteria.

## 1. Introduction

*Capsicum* spp. ranks among the most culturally and economically important horticultural crops worldwide, valued for its sensory diversity, phytochemical richness, and versatility across fresh and processed markets [1,2,3]. Within the genus, five species were domesticated independently: *C. annuum*, *C. chinense*, *C. frutescens*, *C. baccatum*, and *C. pubescens*, reflecting a complex evolutionary history centered in the Andean region and Mesoamerica [4,5,6]. Among them, rocoto (*Capsicum pubescens* Ruiz & Pav.) is distinctive and is cultivated primarily at mid- to high-elevations of the central Andes. The species bears pubescent leaves, purple flowers, and black seeds, and forms a monospecific lineage closely related to *C. cardenasii* and *C. eximium* [7,8,9]. Despite its regional importance in the Andes, *C. pubescens* remains understudied relative to the globally disseminated *C. annuum* complex, and its wild progenitor has not yet been conclusively identified [9,10].

In Peru, rocoto (*Capsicum pubescens*) is a key species within the *Capsicum* complex, with a national production exceeding 200,000 metric tons per year, positioning the country among the world’s leading producers and exporters of the genus *Capsicum*. The crop is cultivated on approximately 14,000 ha by more than 11,000 smallholder farmers, with production mainly concentrated in the Pasco and Junín regions. In recent years, rocoto has strengthened its presence in the export market, reaching up to 19 international destinations; however, production relies largely on poorly improved traditional varieties, which limits its competitiveness. In this context, there is a growing demand for rocoto varieties with improved agronomic traits and higher functional value, aimed at meeting the requirements of export markets and the local agro-processing industry [11,12,13]. Beyond culinary value, rocoto and other chili peppers are a dietary source rich in bioactive compounds with recognized roles in human health and product quality [14,15]. Fruit biochemical profiles typically include high levels of ascorbic acid (vitamin C), diverse phenolics and flavonoids (e.g., quercetin, luteolin), carotenoids, and the phenolic alkaloids known as capsaicinoids [15]. Multi-accession studies and genotype × environment analyses consistently report wide inter- and intraspecific variation in vitamin C, phenolics, and capsaicinoids, underscoring the need to establish species, and region-specific baselines, particularly in underrepresented crops such as rocoto [16,17].

Capsaicinoids, mainly capsaicin and dihydrocapsaicin, are synthesized in placental tissues. Their biosynthesis is regulated by ontogenic signals that emerge during fruit development and are modulated by genetic factors, environmental conditions such as temperature, light, water availability, and their interaction [18,19]. The species-level biochemical signature of *C. pubescens* is characterized by a dihydrocapsaicin-dominant profile, in contrast to the capsaicin-predominant pattern seen in *C. chinense* and *C. frutescens* [20,21]. Such interspecific differences may influence perceived pungency, antioxidant activity, and potential health-related properties, reinforcing the importance of species-specific phytochemical benchmarks for breeding and the development of high-value products [22].

Standardized fruit descriptors (size, shape, pericarp thickness, seed variables) are directly linked to commercial classification and processing behavior, while color, quantified in a device-independent CIELAB space, constitutes an essential quality attribute for acceptance in the fresh market and for stability in dried or milled products [23,24,25].

Although genetic and genomic studies have clarified aspects of *C. pubescens* diversification [9,26], a major knowledge gap persists regarding the relationships between agro-morphological diversity and functional phytochemical variables within the species, particularly in germplasm cultivated in its center of origin. Strategically, closing this gap could accelerate pre-breeding, inform selection indices that integrate market and nutritional quality, and guide innovation along high-Andean horticultural value chains.

To address this need, we conducted a comprehensive morpho-functional profiling of 78 *C. pubescens* accessions from the INIA (Peru) Rocoto Germplasm Collection. Specifically, we (i) evaluated agro-morphological variables relevant to commercial classification and processing, including plant height, days to fruit maturity, fruit yield per plant, CIELAB color, fruit weight, seed weight per fruit, edible portion weight, fruit dimensions, and number of locules; (ii) quantified physicochemical variables comprising soluble solids and fruit moisture content; and (iii) determined functional phytochemical variables, including total phenolics, total carotenoids, ascorbic acid, antioxidant activity, and capsaicinoids. By integrating these domains, this study establishes robust, species-specific references for rocoto, identifies superior phenotypes that combine desirable morphology with enhanced functional quality, and provides actionable targets for breeding programs and the development of high-value products with nutraceutical potential.

## 2. Materials and Methods

### 2.1. Plant Material

**Figure 1:**
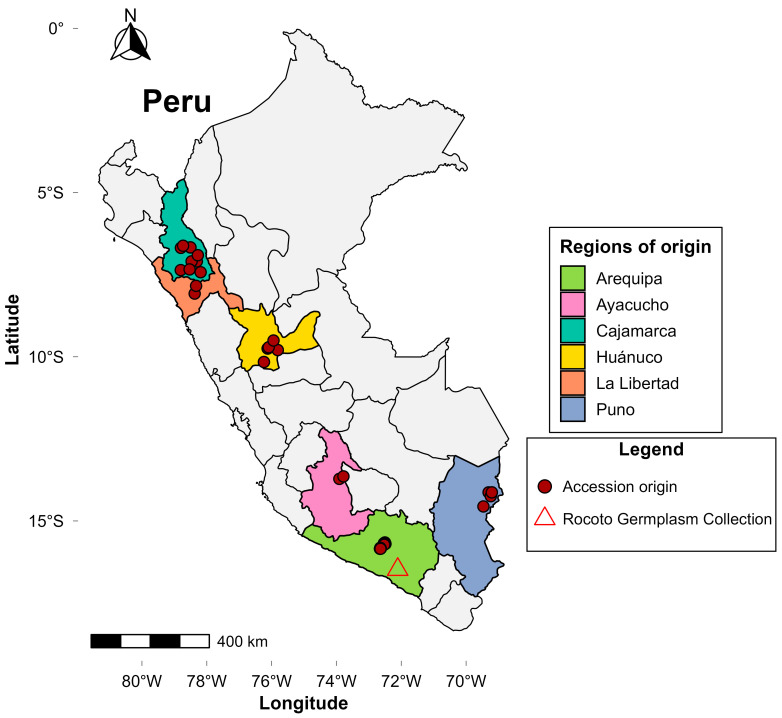
Geographic origin of *Capsicum pubescens* Ruiz & Pav. accessions from the Rocoto Germplasm Collection, conserved at INIA’s EEA Arequipa, Peru.

Seventy-eight *Capsicum pubescens* accessions originating from six Peruvian regions (Arequipa, Ayacucho, Cajamarca, Huánuco, La Libertad, and Puno; see Figure 1) were evaluated; these were collected as part of the National Rocoto Germplasm Collection of the Instituto Nacional de Innovación Agraria (INIA). All accessions correspond to germplasm materials maintained as open-pollinated seed lots in the collection. Field trials were established at the Estación Experimental Agraria Arequipa—Centro Experimental Santa Rita (1296 m a.s.l.; 16°28′19″ S, 72°06′36″ W), located in the Santa Rita de Siguas district, Arequipa Department (Figure 1). The site has an arid climate (mean annual temperature, 19.2 °C; mean relative humidity, 28.7%; see Table S5 for detailed climatic data), a loamy-sand soil texture, electrical conductivity of 93.7 mS/m (normal range), available phosphorus of 8.2 mg/kg (medium), and low organic matter (0.9%). Fertilization was applied at rates of 200 kg N ha^−1^, 150 kg P_2_O_5_ ha^−1^, 350 kg K_2_O ha^−1^, 100 kg CaO ha^−1^, and 80 kg Mg ha^−1^, with fertigation managed through a drip irrigation system according to the program detailed in Table S4. The evaluation plot measured 12.24 m^2^ (7.2 m× 1.7 m), with 1.8 m spacing between plants. Each accession was established with five plants arranged in a single row. Sampling was conducted during the first harvest of the 2024 season (October–November). For each accession, 20 ripe fruits, which showed visible physiological maturity, were collected from the three central plants to avoid border effects. The fruits were used for morphological characterization at EEA Arequipa and for phytochemical analysis at the Laboratorio de Investigación Nutricional de los Recursos Genéticos at INIA’s central headquarters.

### 2.2. Reagents, Chemicals, and Sample Preparation

Analytical-grade reagents included Folin–Ciocalteu reagent, sodium carbonate, butylated hydroxytoluene (BHT), petroleum ether, dinitrophenylhydrazine–thiourea–copper sulfate (DTC), potassium persulfate, ferric chloride, sodium acetate, glacial acetic acid, 2,2′-azino-bis(3-ethylbenzothiazoline-6-sulfonic acid) diammonium salt (ABTS), 2,2-diphenyl-1-picrylhydrazyl (DPPH), 2,4,6-tri(2-pyridyl)-s-triazine (TPTZ), and the HPLC-grade solvents acetonitrile and ethanol; all were purchased from Merck, Sigma-Aldrich, and J.T. Baker (Phillipsburg, NJ, USA). Quantification standards included gallic acid, β-carotene, nordihydrocapsaicin, capsaicin, dihydrocapsaicin, and Trolox (Merck, Sigma-Aldrich, St. Louis, MO, USA). Type I water was produced with a MicroPure ST purification system (Thermo Scientific, Darmstadt, Germany). Rocoto fruits were cleaned and manually de-stemmed.

Ten fruits per accession were selected for morphological characterization and the remaining ten for phytochemical analyses. These fruits were subjected to a sun-drying method described by Rochín-Wong et al. [27], with modifications. They were placed on stainless-steel trays and dried inside a screenhouse, which provided adequate ventilation and protection from direct solar radiation. To prevent overheating and ensure uniform drying, the upper trays were not used. These conditions minimized photodegradation and helped preserve the integrity of the plant material for subsequent analyses at EEA Arequipa. Under these conditions, temperatures ranged from 24 to 40 °C, with an average relative humidity of approximately 40% for five days (≈40 h effective). Dried samples were milled, packed in laminated bags, and stored in the dark at room temperature until analysis.

### 2.3. Agro-Morphological Characterization

Agro-morphological descriptors were assessed following the *Capsicum* spp. descriptor of the International Plant Genetic Resources Institute (IPGRI) [28]: plant height (PH), days to maturity (MD), fruit weight per plant (FWP), fruit weight (WEI), fruit dimensions (FL, length; FW, width; FT, pericarp thickness), and number of locules (LN). Seed weight per fruit (FSW) was determined with a precision balance (Radwag, WTC 2000, Radom, Poland). Edible portion weight (EFW, pericarp + placenta) was calculated as total fruit weight minus seed weight per fruit. Fruit color variables—L, lightness (L*); A, redness (a*); B yellowness (b*); C, chroma (C*); and H, hue angle (*h°*)—were measured using a portable colorimeter (Konica Minolta CR-400, Tokyo, Japan) calibrated with a standard white tile [29]. Measurements were taken directly on the skin surface, with two readings on the equatorial plane for each fruit. Agronomic descriptors were evaluated in five plants, and morphological descriptors were assessed in ten fruits.

### 2.4. Physicochemical Variables

Soluble solids (BRIX, Santiago, Chile) in fresh fruit were determined according to AOAC 920.149 and AOAC 932.12-1980 [30,31] and expressed as °Brix. Pulp was homogenized by grinding and filtered through Whatman No. 4 paper; readings were taken on a digital refractometer (Hanna Instruments HI96801, Woonsocket, RI, USA). Fruit moisture content (HUM) was evaluated by the gravimetric method (AOAC 930.15) [32], with minor modifications. For this, 5 g of homogenized pulp were placed in an oven at 105 °C until a constant weight was reached. Moisture content was calculated as the difference between the initial weight of the fresh sample and the final dry weight and expressed as a percentage of the fresh sample. Physicochemical variables were assessed in ten fruits per triplicate. Based on the measured moisture content, functional compounds quantified in subsequent analyses (total phenolics, carotenoids, ascorbic acid, antioxidant activity and capsaicinoids) were expressed on a dry-weight (DW) basis. Fresh-weight (FW) equivalents can be derived when required using the moisture content (FW = DW × (1 − HUM/100)).

### 2.5. Bioactive Compounds and Antioxidant Activity

#### 2.5.1. Total Phenolic Content

Extraction followed Meckelmann et al. [17], with minor modifications: 100 mg of dried sample were combined with 0.5 mL sodium phosphate buffer (0.5 M, pH 11) and 7.5 mL acetonitrile/methanol (50:50, *v*/*v*); incubation proceeded for 16 h at 4 °C in the dark, followed by 4 h at 80 °C with vortexing every 30 min (Vortex-Genie 2, G560E, Bohemia, NY, USA). The crude extract was diluted 1:1 with methanol/water (1:1, *v*/*v*), homogenized, and centrifuged (Eppendorf 5430 R, Hamburg, Germany) at 4 °C for 30 min at 7400 rpm. The supernatant was transferred to polyethylene tubes. Total phenolic content (TPC) was quantified via the Folin–Ciocalteu procedure (Meckelmann et al. [17], with adjustments): 0.1 mL extract + 0.9 mL distilled water + 5 mL Folin/water (1:10, *v*/*v*); after 5 ± 2 min at 30 °C, 4 mL sodium carbonate solution (7.5 g/100 mL) were added; after 1 h at 30 °C, absorbance was read at 750 nm on a UV–Vis spectrophotometer (Thermo Scientific GENESYS 150) using a quartz cuvette. A gallic acid calibration curve (60–520 mg/L) was used.

#### 2.5.2. Total Carotenoids

Following Neitzke et al., [33], with modifications, 0.4 g of sample were extracted with 5 mL acetone/ethanol (1:1, *v*/*v*) containing BHT (200 mg/L); samples were centrifuged at 7400 rpm for 10 min at 4 °C and extracted four times to a final volume of 20 mL. Petroleum ether (10 mL) and distilled water (5 mL) were added to the extract, vigorously shaken, and phases allowed to separate. Total carotenoid content (CAR) was quantified by measuring the absorbance of the organic phase at 450 nm using spectrophotometry.

#### 2.5.3. Ascorbic Acid

According to Al-Ani et al. [34], 1 g of sample was diluted with metaphosphoric acid (6 g/dL) to 25 mL, centrifuged at 7400 rpm for 15 min at 4 °C, and filtered (0.45 μm). To 1.2 mL of filtrate, 0.4 mL of DTC reagent were added; the mixture was incubated for 3 h at 37 °C, cooled for 10 min in a thermostatic water bath (Memmert WNE 14, Schwabach, Germany), then 2 mL of H_2_SO_4_ (12 mol/L) were added. Ascorbic acid content (ASC) was quantified by spectrophotometric measurement at 520 nm.

#### 2.5.4. Antioxidant Activity

The extract from the TPC assay was used. ABTS followed Hervert-Hernández et al. [35], with adjustments: the ABTS•^+^ cation was generated by reacting ABTS (7 mM) with potassium persulfate (2.45 mM) 16 h prior in the dark; the solution was diluted with methanol to A = 0.70 ± 0.02 at 730 nm; 0.1 mL extract + 3.9 mL ABTS were reacted for 6 min and read at 730 nm. DPPH followed Medina-Juárez et al. [36]: 3.9 mL DPPH (0.06 mM, methanolic) + 0.1 mL extract; 30 min in the dark; reading at 515 nm.

FRAP was adapted from Hervert-Hernández et al. [35], with modifications: the FRAP reagent (acetate buffer 300 mM, pH 3.6; TPTZ 10 mM in 40 mM HCl; FeCl_3_·6H_2_O 20 mM; 10:1:1) was preheated to 37 °C; 0.12 mL extract + 0.36 mL methanol/water (1:1, *v*/*v*) + 3.6 mL FRAP were mixed, incubated 4 min, and read at 595 nm.

### 2.6. HPLC Analysis of Capsaicinoids and Pungency

Extraction and quantification followed the American Spice Trade Association (ASTA) method 21.3 [37], with modifications. For extraction, 50 mg of sample were combined with 10 mL ethanol (95%, *v*/*v*), vortexed vigorously, and incubated for 1 h (timed from the onset of boiling) in a thermostatic water bath at 75 ± 5 °C; the extract was cooled to room temperature and filtered (0.45 μm). One milliliter was injected into an HPLC system (Waters e2695 Alliance, Miami, FL, USA) with fluorescence detection (λ_ex = 280 nm; λ_em = 320 nm). Separation used a LiChrospher^®^ 100 RP-18 column (4.6 mm × 150 mm, 5 μm; Merck KGaA, Darmstadt, Germany). Temperatures were 45 °C (column) and 12 °C (autosampler). Mobile phases were Type I water with 1% acetic acid (*v*/*v*) and acetonitrile (60:40, isocratic), at 1.0 mL/min; injection volume was 20 μL. Retention times were 16 min for nordihydrocapsaicin (NHC), 18 min for capsaicin (CAP), and 28 min for dihydrocapsaicin (DHC). Baseline separation was achieved under the applied isocratic conditions, corresponding to a chromatographic resolution (Rs) ≥ 1.5. A representative HPLC chromatogram of a standard solution is shown in Technical Report S1, clearly demonstrating the adequate separation of the capsaicinoids. Quantification used calibration curves (0.15–1.0 mg/L), and results were expressed as μg/g. Total capsaicinoid content (TCS) was calculated as sum of the three major capsaicinoids.

For method validation, the limits of detection (LOD) and quantification (LOQ) were estimated based on the repeatability data obtained from fortified blanks (*n* = 7), analyzed over three different days, were calculated based on the standard deviation of the response and the slope following the equations: LOD = 3Q/S; LOQ = 10Q/S, where Q is the standard deviation of the response, and S is the slope of the calibration curve, in this condition LOD was 0.0472 μg/g for nordihydrocapsaicin, 0.0472 μg/g for capsaicin and 0.0473 μg/g dihydrocapsaicin. LOQ was 0.1501 μg/g, 0.1502 μg/g and 0.1506 μg/g for nordihydrocapsaicin, capsaicin and dihydrocapsaicin, respectively. The precision was evaluated by RSD (Relative Standard Deviation) of peak area and retention time, using solution standard in three levels of concentration. Repeatability (Intra-day precision) was determined by 10 injections on the same day, and reproducibility (Inter-day precision).

Pungency was expressed in Scoville Heat Units and determined according to the American Spice Trade Association (ASTA) method 21.3 [37].

### 2.7. Statistical Analysis

All analyses were performed in RStudio 2025.09.0 + 387 and GraphPad Prism 10.6. First, univariate descriptive statistics (mean, standard deviation, coefficient of variation, kurtosis, skewness) were computed to characterize the distribution and phenotypic range of 28 quantitative variables (agro-morphological variables, CIELAB color, physicochemical variables, and bioactive compounds). Pairwise Spearman correlations were computed among the 24 quantitative traits. To account for multiple testing, *p*-values were adjusted using the Benjamini–Hochberg false discovery rate (FDR) procedure, and unless otherwise stated, significance refers to FDR-adjusted *p*-values. The Spearman correlation matrix was constructed using the corrplot and MVN packages and visualized as a triangular heat map with color coding and significance levels to facilitate modular interpretation and detection of cross-block associations. To explore multivariate structure across the 78 accessions, principal component analysis (PCA) was conducted on standardized data (centered and scaled) using readr, dplyr, ggplot2, ggrepel, and factoextra. Prior to PCA, sampling adequacy was assessed using the Kaiser–Meyer–Olkin (KMO) measure and Bartlett’s test of sphericity applied to the correlation matrix. Two analytical configurations were implemented: (i) chromatic + agro-morphological and (ii) chromatic + physicochemical. In addition, hierarchical cluster analysis (HCA) was performed using Euclidean distance and Ward.D2 agglomeration on standardized data; groups were defined based on dendrogram height and phenotypic interpretation and projected onto the PCA plane. In addition, hierarchical cluster analysis (HCA) was performed using Euclidean distance and Ward.D2 agglomeration on standardized data. The optimal number of clusters (K) was evaluated by average silhouette width for K values from 2 to 6, using the cluster and factoextra packages. The solution with K = 3 showed the highest average silhouette width and yielded biologically interpretable groups, and was therefore retained for subsequent interpretation. These analyses employed agricolae, factoextra, ggplot2, RColorBrewer, dendextend, circlize, ape, and grid. For group comparisons by zone of origin, we applied Kruskal–Wallis rank-sum tests, followed, when appropriate, by Dunn’s post hoc test with multiplicity adjustment (Holm/Bonferroni). Visualizations included boxplots (mean ± SE) and violin plots according to data nature/distribution, generated in GraphPad Prism 10.6. Finally, a multi-trait selection index was developed based on 12 agronomically and functionally relevant variables (MD, FWP, FL, FW, WEI, a*, TPC, ASC, CAR, FRAP, TCS, and SHU). Each accession received a standardized score from 1 to 6 per trait (6 = most favorable), with the scale inverted for MD to favor earliness. Each of the 12 target traits was converted into a standardized 1–6 score using a quantile-based ranking scheme. For a given trait, accession values were sorted and partitioned into six equally sized groups (approximately sextiles), assigning scores from 1 (least desirable) to 6 (most desirable). For MD (days to maturity), the scoring was reversed so that lower MD values receive higher scores, reflecting the preference for earlier maturity. The overall multi-trait selection score was computed as the sum of the 12 trait scores (range 12–72), and accessions with a total score >48 were considered promising, whose 8 top superior phenotype profiles were plotted as per-trait bar charts in GraphPad Prism 10.6. The geographic map was generated with dplyr, ggplot2, sf, rnaturalearth, ggspatial, RColorBrewer, cowplot, and grid, integrating spatial coordinates and graphical customization.

## 3. Results

### 3.1. Agro-Morphological, Physicochemical, Bioactive Compounds, and Antioxidant Characterization

Table 1 synthesizes phenotypic variability across 78 rocoto accessions, encompassing agro-morphological, physicochemical, and functional quality variables. Within the vegetative–productive component, plant height (PH) averaged 92.65 cm (CV = 9.76%, 64.33–108.70 cm) and days to maturity (MD) averaged 247.60 days (CV = 6.76%, 207–296 days), both displaying moderate skewness and suitable behavior for parametric analyses. Fruit weight per plant (FWP) exhibited pronounced heterogeneity (mean = 510.10 g; CV = 91.04%, 12.69–1838 g) with a right-skewed distribution, revealing a subset of exceptionally productive accessions at the upper tail. The wide range observed for FWP reflects the presence of distinct low- and high-yielding accession groups within the panel rather than isolated outlier values.

Color spanned a broad, and generally non-normal (*p* < 0.001), range consistent with the chromatic diversity of rocoto. Mean lightness (L*) was 46.63 (CV = 20.08%, 33.73–59.11), redness (a*) 26.24 (CV = 25.05%, 14.25–37.52), yellowness (b*) 37.52 (CV = 41.97%, 16.77–74.39), chroma (C*) 47.73 (CV = 17.95%, 32.45–62.14), and hue angle (*h°*) 51.69 (CV = 35.00%, 26.48–73.45). The largest CV in b* (~42%) reflects marked heterogeneity along the yellow–blue axis; negative kurtosis and skewness indicate relatively flat distributions with light tails, as is typical for color traits varying along that axis. For fruit morphology, individual mass and dimensions showed low-to-moderate variation and near-normal distributions. Pericarp thickness (FT) averaged 3.96 mm (CV = 10.12%, 3.11–4.77 mm), and individual fruit weight (WEI) averaged 24.58 g (CV = 23.65%, 11.50–40.31 g). Seed weight per fruit (FSW) was 0.70 g (CV = 29.03%), and edible portion weight (EFW) 23.88 g (CV = 23.97%), mirroring these patterns. Fruit length (FL) averaged 4.70 cm (CV = 18.14%, 2.87–6.69 cm) and width (FW) 3.76 cm (CV = 11.92%, 2.63–4.73 cm). The number of locules (LN) averaged 2.74 (CV = 13.99%) with acceptable normality, suggesting variation in compartmentalization without extremes.

Fresh-fruit quality was defined by soluble solids and fruit moisture content. Soluble solids averaged 8.46% (CV = 11.13%, 6.60–11.40%) with a distribution not significantly different from normal; fruit moisture content (HUM) was high (89.41%) and very uniform (CV = 1.26%) with only slight deviation from normality. Thus, while some accessions have higher soluble solids—directly associated with perceived sweetness—water content in fresh fruit is consistently high, as expected for a vegetable crop. For functional quality, total phenolics (TPC) averaged 1382 mg GAE/100 g (CV = 18.28%, 945–2317 mg GAE/100 g) with right-skewed distribution. Carotenoids (CAR) averaged 19.62 mg β-carotene equivalents/100 g with a very high CV (57.0%, 2.50–52.59 mg β-carotene eq/100 g); ascorbic acid (ASC) reached 70.44 mg/100 g (CV = 26.36%, 34.35–128 mg/100 g) with a clear upper tail. Antioxidant capacity spanned wide ranges: ABTS 113.30 μmol TE/g (CV = 33.70%), DPPH 16.82 μmol TE/g (CV = 41.72%), and FRAP 54.93 μmol TE/g (CV = 17.00%), the latter showing non-normality and positive skew, indicating a subset of promising accessions with elevated antioxidant activity and phenolics. For capsaicinoids, means were NHC 486.40 μg/g (CV = 52.03%), CAP 802.50 μg/g (CV = 52.26%), and DHC 871.90 μg/g (CV = 49.38%); total capsaicinoids (TCS) reached 2161 μg/g (CV = 45.78%) and pungency (SHU) 34,509 (CV = 46.60%, 6814–83,961), all with slight right tails that help pinpoint elite high-heat accessions within the germplasm.

### 3.2. Correlation Analysis

**Figure 2:**
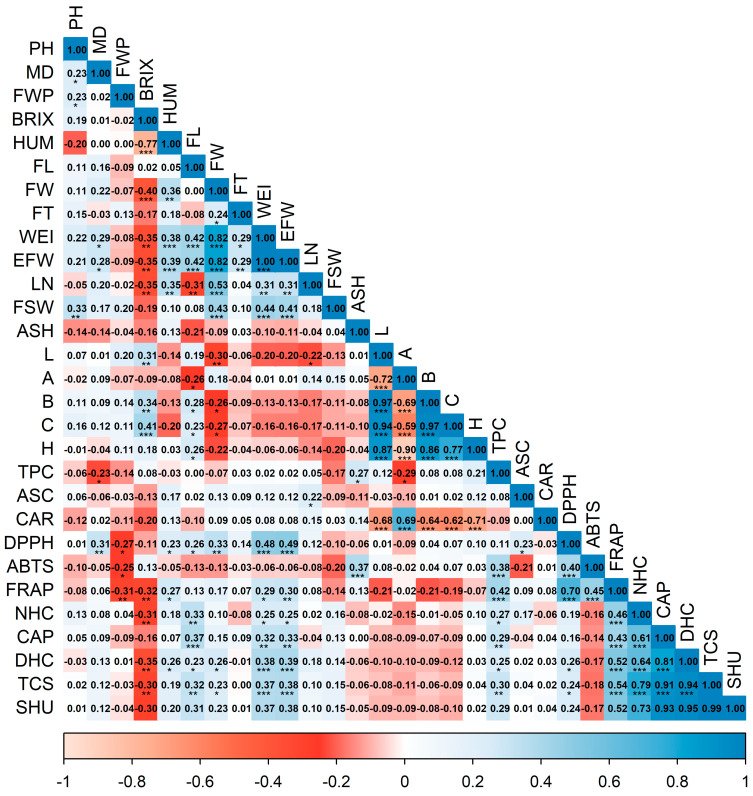
Triangular heatmap of pairwise Spearman correlations among agro-morphological, physicochemical, bioactive, and antioxidant variables in 78 rocoto accessions. Abbreviations: PH, plant height; MD, days to maturity; FWP, fruit weight per plant; BRIX, soluble solids; HUM, fruit moisture content; FL, fruit length; FW, fruit width; FT, pericarp thickness; WEI, individual fruit weight; EFW, edible fruit weight; LN, number of locules; FSW, seed weight per fruit; L, fruit lightness (L*); A, red–green coordinate (a*); B, yellow–blue coordinate (b*); C, chroma (C*); H, hue angle (*h°*); TPC, total phenolic content; ASC, ascorbic acid; CAR, total carotenoids; DPPH, DPPH antioxidant assay; ABTS, ABTS antioxidant assay; FRAP, FRAP antioxidant assay; NHC, nordihydrocapsaicin; CAP, capsaicin; DHC, dihydrocapsaicin; TCS, total capsaicinoids; SHU, Scoville heat units. Significance: * p < 0.05; ** *p* < 0.01; *** *p* < 0.001. All *p*-values were corrected by Benjamini–Hochberg FDR (α = 0.05).

The Spearman correlation matrix (Figure 2) revealed a recognizable pattern with three visually coherent blocks emerging from the ordering of variables: an agro-morphological block, a color block, and a functional-quality block. Within the morphological component, fruit length covaried highly significantly (*p* < 0.001) and positively with both individual fruit weight and edible portion weight, sharing the same correlation coefficient (FL–EFW, 0.42; FL–WEI, 0.42), and was negatively associated with the number of locules (FL–LN, −0.31, *p* < 0.01).

Individual fruit weight correlated significantly with days to maturity (WEI–MD, 0.29, *p* < 0.05), highly significantly with fruit length (WEI–FL, 0.42, *p* < 0.001), and even more strongly with fruit width (EFW–FW, 0.82, *p* < 0.001); by contrast, it was negatively associated with soluble solids (WEI–BRIX, −0.35, *p* < 0.01). Edible fruit weight showed a significant positive correlation with days to maturity (EFW–MD, 0.28, *p* < 0.05), a highly significant correlation with fruit moisture content (EFW–HUM, 0.39, *p* < 0.001), and a very significant correlation with pericarp thickness (EFW–FT, 0.29, *p* < 0.01); in contrast, it was very significantly and negatively associated with soluble solids (EFW–BRIX, −0.35, *p* < 0.01). The number of locules correlated moderately and positively with fruit width (LN–FW, 0.53, *p* < 0.001) and moderately with fruit moisture content (LN–HUM, 0.35, *p* < 0.01); it also showed moderate negative associations with soluble solids (LN–BRIX, −0.35, *p* < 0.01) and fruit length (LN–FL, −0.31, *p* < 0.01).

Seed weight per fruit correlated positively with plant height (FSW–PH, 0.33, *p* < 0.001), individual fruit weight (FSW–WEI, 0.44, *p* < 0.001), and edible fruit weight (FSW–EFW, 0.41, *p* < 0.001). Pericarp thickness correlated positively only with fruit width (FT–FW, 0.24, *p* < 0.05) and displayed weak, non-significant correlations with the remaining morphological variables, suggesting that pericarp thickness varies largely independently.

The CIELAB color variables were strongly intercorrelated (*p* < 0.001). Lightness decreased as redness increased (L–A, −0.72) and was positively related to yellowness (L–B, 0.97), chroma (L–C, 0.94), and hue (L–H, 0.87). Redness was negatively related to yellowness (A–B, −0.69), chroma (A–C, −0.59), and hue (A–H, −0.90). Chroma and hue were positively associated (C–H, 0.77), and yellowness aligned with both (B–C, 0.97; B–H, 0.86), defining a clear–yellow–saturated gradient that contrasts with the direction of redness.

Fresh-market quality traits integrated logically into this chromatic gradient. Soluble solids (°Brix) correlated positively with lightness, yellowness, and chroma (BRIX–L, 0.31, *p* < 0.01; BRIX–B, 0.34, *p* < 0.01; BRIX–C, 0.41, *p* < 0.001), were not significantly related to hue (BRIX–H, 0.18, *p* > 0.05), and were negatively associated with redness (BRIX–A, −0.09, *p* > 0.05). Conversely, °Brix decreased with increasing mass (BRIX–WEI/EFW, −0.35, *p* < 0.01) and with the number of locules (BRIX–LN, −0.35, *p* < 0.01), and showed a marked antagonism with fruit moisture content (BRIX–HUM, −0.77, *p* < 0.001), while moisture related positively and moderately to weight (HUM–WEI, 0.38, *p* < 0.001). Physiologically, heavier fruits contain more water and therefore lower sugar concentrations, explaining why the sweetest accessions also tend to occupy the light/yellow/saturated octant of CIELAB space.

Two functional submodules emerged. The phenolic–antioxidant submodule exhibited moderate couplings consistent with significant partial contributions of the phenolic pool to redox capacity (FRAP–TPC, 0.42; ABTS–TPC, 0.38). Antioxidant assays were positively intercorrelated, with highly significant associations (*p* < 0.001) for ABTS–FRAP (0.45) and ABTS–DPPH (0.40), and a strong linkage for DPPH–FRAP (0.70). Ascorbic acid showed low correlations with most variables except DPPH (ASC–DPPH, 0.23, *p* < 0.05), and a weak, non-significant positive correlation with total phenolics (ASC–TPC, 0.08, *p* > 0.05).

The capsaicinoid/pungency submodule was highly cohesive. Positive, highly significant correlations (*p* < 0.001) were observed among capsaicinoids (CAP–NHC, 0.61; NHC–DHC, 0.64; CAP–DHC, 0.81), highlighting a strong CAP–DHC association, which in turn explained the high correlations with total capsaicinoids (TCS–CAP, 0.91; TCS–DHC, 0.94; TCS–NHC, 0.79). Pungency depended primarily on dihydrocapsaicin and capsaicin (SHU–DHC, 0.95; SHU–CAP, 0.93; SHU–NHC, 0.73; all *p* < 0.001).

Cross-block correlations yielded operational cues for selection. First, although small in magnitude, several associations between pungency and morphology were significant: SHU correlated positively with fruit length (SHU–FL, 0.31, *p* < 0.01), fruit width (SHU–FW, 0.23, *p* < 0.05), individual fruit weight (SHU–WEI, 0.37, *p* < 0.001), and edible fruit weight (SHU–EFW, 0.38, *p* < 0.001). At the population level, these statistically significant but low-magnitude correlations should be interpreted as indicative trends rather than direct determinants of capsaicinoid biosynthetic capacity at the individual accession level. Second, a weak but significant link was detected between pungency and total phenolics (SHU–TPC, 0.29, *p* < 0.05), accompanied by positive correlations between pungency and antioxidant capacity (SHU–FRAP, 0.52, *p* < 0.001), opening the possibility of profiling products that are simultaneously hot and antioxidant-rich. Third, ascorbic acid showed a weak association with the number of locules (ASC–LN, 0.22, *p* < 0.05), while carotenoids correlated significantly and positively with redness (CAR–A, 0.69, *p* < 0.001) and strongly and negatively with yellowness, chroma, lightness, and hue (CAR–L, −0.68; CAR–B, −0.64; CAR–C, −0.62; CAR–H, −0.71; all *p* < 0.001), suggesting an association between red coloration and higher carotenoid content.

### 3.3. Principal Component Analysis

**Figure 3:**
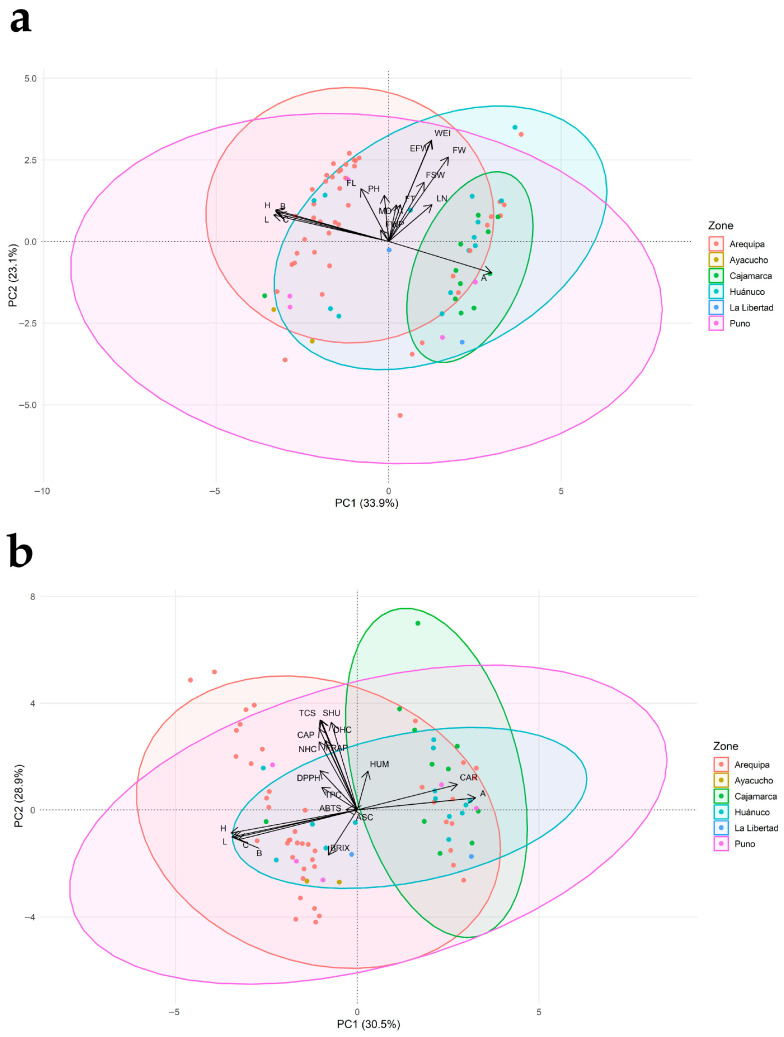
Principal component analysis (PCA) of 78 rocoto accessions based on agro-morphological, chromatic, and functional variables. (**a**) Relationship between fruit color variables and agro-morphological variables. (**b**) Relationship between fruit color variables, physicochemical and functional compounds. Colors indicate each accession’s zone of origin. Abbreviations: PH, plant height; MD, days to maturity; FWP, fruit weight per plant; BRIX, soluble solids; HUM, fruit moisture content; FL, fruit length; FW, fruit width; FT, pericarp thickness; WEI, individual fruit weight; EFW, edible portion weight; LN, number of locules; FSW, seed weight per fruit; L, lightness L*; A, color coordinate a*; B, color coordinate b*; C, chroma C*; H, hue angle *h°*; TPC, total phenolic content; ASC, ascorbic acid content; CAR, total carotenoids; DPPH, DPPH antioxidant assay; ABTS, ABTS antioxidant assay; FRAP, FRAP antioxidant assay; NHC, nordihydrocapsaicin; CAP, capsaicin; DHC, dihydrocapsaicin; TCS, total capsaicinoids; SHU, Scoville heat units.

Figure 3 presents two PCA biplots for the 78 *Capsicum pubescens* accessions, differentiated by provenance. Data suitability for PCA was supported by an overall KMO of 0.68 (acceptable sampling adequacy) and a highly significant Bartlett’s test of sphericity (χ^2^ = 2346.24; df = 105; *p* < 0.001), indicating sufficient correlation structure to justify dimensionality reduction. Full outputs are provided in Annex S1. Panel (a) explores relationships between agro-morphological attributes and fresh-fruit color variables, whereas panel (b) examines the association between functional compounds and color variables.

In panel (a), PC1 explained 33.9% of the variance and was strongly influenced by fruit size and weight (FW, WEI, EFW), pericarp thickness (FT), and number of locules (LN), while PC2 (23.1%) captured the main variation in plant height (PH), fruit length (FL), and the chromatic variables L*, b*, C*, and *h°*. Vectors associated with fruit size and weight (FW, WEI, EFW) point in the same direction, indicating positive correlations among these variables, while b* opposes a*, confirming the strong antagonism between the yellow–orange coordinate and redness, as also reflected in the correlation matrix. The dispersion of accessions suggests that those from Huánuco and Cajamarca tend to cluster on the right semiplane, where larger size and redness coincide, whereas accessions from Puno and Arequipa spread toward regions associated with more elongated fruits and greater chromatic variability.

Panel (b), the principal component analysis integrating color parameters with phytochemical variables explains nearly 60% of the total variation in the first two components (PC1 = 30.5%, PC2 = 28.9%). PC1 defines a clearly functional gradient: towards positive values, the vectors corresponding to total phenolic content (TPC), antioxidant capacity assessed by FRAP and ABTS, ascorbic acid content (ASC), total carotenoids (CAR), individual capsaicinoids (NHC, CAP, and DHC), total capsaicinoid content (TCS), and pungency expressed in Scoville Heat Units (SHU) are projected. This clustering of vectors indicates that, in the accessions evaluated, higher antioxidant capacity and higher capsaicinoid content tend to co-occur within the same set of genotypes. On the opposite side of PC1, the CIELAB color parameters (L*, a*, b*, C*, and *h°*) are located, revealing that the color coordinates show only weak or negative associations with most of the quantified bioactive compounds, such that external fruit color alone is not a good predictor of internal functional content. PC2, in turn, primarily separates the pungency/capsaicinoid axis—whose vectors point toward positive PC2 values—from the color parameters, which are located toward negative values of this component, and from the hydrophilic antioxidant variables, which occupy intermediate positions. This pattern suggests that variation in pungency is not always accompanied by proportional changes in antioxidant capacity as measured by FRAP and ABTS. Fruit moisture content is positioned close to the origin, indicating a weak relationship with most functional traits, whereas soluble solids show a moderate contribution to PC1 and PC2, consistent with the notion that fruit sweetness is largely decoupled from the content of the bioactive compounds evaluated. Regarding the distribution of accessions by region of origin, the ellipses for Cajamarca and Arequipa are shifted mainly toward the quadrants with positive PC1 values (and, in the case of Cajamarca, also positive PC2 values), indicating a higher frequency of genotypes with elevated phenolic, carotenoid, and capsaicinoid contents and high pungency. Accessions from Huánuco occupy an intermediate position, combining moderate levels of functional compounds with considerable within-region dispersion, whereas those from Ayacucho and Puno are concentrated mostly toward negative PC1 values, reflecting more modest phytochemical profiles, although with some isolated genotypes projecting into the quadrants associated with higher carotenoid or capsaicinoid content.

### 3.4. Hierarchical Clustering

**Figure 4:**
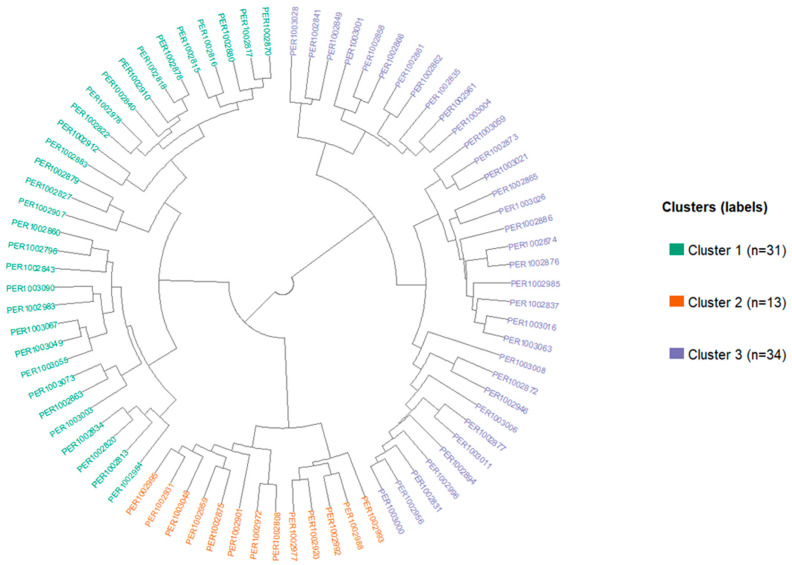
Circular dendrogram from hierarchical clustering of 78 rocoto accessions based on agro-morphological, chromatic, and functional variables.

**Figure 5:**
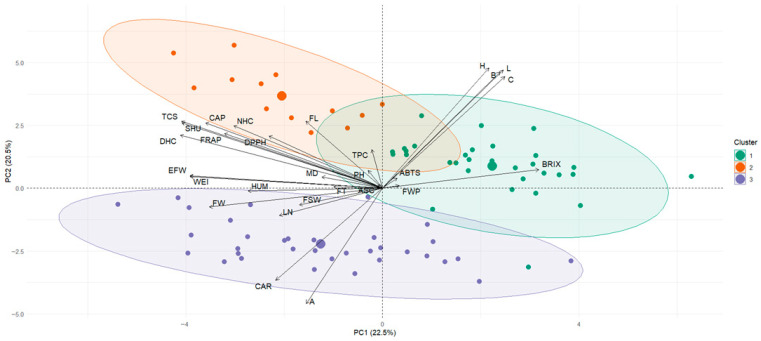
PCA biplot of 78 rocoto accessions, colored by hierarchical cluster, with variable loadings (agro-morphological variables, color, and functional variables). Explained variance: PC1 = 22.5%, PC2 = 20.5%.

Three phenotypic groups were identified through hierarchical clustering (Figure S1) in the panel of 78 accessions, hereafter referred to as Cluster 1 (*n* = 31), Cluster 2 (*n* = 13), and Cluster 3 (*n* = 34). The circular dendrogram (Figure 4) displays the three groups recovered by the clustering analysis, with branches that visually separate each cluster, and projecting the accessions onto the PCA biplot (Figure 5) corroborates their separation (PC1 = 22.50%, PC2 = 20.50%), with each cluster occupying a characteristic sector. The coverage ellipses show only slight overlaps, indicating internal coherence. Cluster 1 lies predominantly on the right half-plane of the biplot, aligned with fresh-fruit color vectors other than redness (L*, b*, C*, *h°*) and with soluble solids. Practically, it groups materials bearing visually lighter, yellower, and more saturated fruits with higher sweetness; at the same time, it exhibits lower fruit moisture content and lower individual fruit weight than the groups projecting toward negative PC1, consistent with the soluble solids–moisture antagonism and the physiological dilution gradient. Within the functional quality block, ABTS, and to a lesser extent total phenolics (TPC), tracks the orientation of Cluster 1, suggesting that part of this cluster combines chromatic versatility for the fresh market with antioxidant response captured by ABTS. By contrast, plant-level and yield-per-plant traits (PH, MD, FWP) contribute little to the separation of Cluster 1 in the first two components, indicating that this group’s identity is dominated by table-quality attributes, with color diversity and alternatives to what currently prevails in the market.

Cluster 2 occupies the upper half-space (positive PC2), projecting to the left, and aligns with the capsaicinoid/pungency core (CAP, DHC, TCS, SHU) together with antioxidant activity (FRAP and DPPH). This pattern reveals accessions with high pungency and high reducing capacity (most clearly captured by FRAP), consistent with the moderate-to-strong correlations between these assays and pungency status. The FL vector also points toward this cluster’s sector, in line with weak associations between fruit length and pungency, suggesting that slightly longer fruits contribute to higher pungency in this set.

Cluster 3 projects into the lower-left quadrant (negative PC1 and PC2) and groups accessions with greater fruit mass and water content; the WEI, EFW, FW, FSW, HUM, and, to a lesser extent, LN vectors point toward its ellipse. Opposite to Cluster 1, Cluster 3 concentrates materials with lower soluble solids and fresh-fruit color that is less light/yellow/saturated. Notably, carotenoids load in the same quadrant, indicating that in this panel carotenoid accumulation is partially decoupled from the sweetness gradient and from the non-red chromatic system, an advantageous feature for both markets and breeding, and relates more closely to the mass/moisture package. This configuration is consistent with the weak cross-block correlations observed between carotenoids and soluble solids/non-red color in the correlation analysis.

Taken together, the PCA anchored on the three clusters outlines three functional phenotypic profiles with weak cross-block correlations across axes: (i) Cluster 1, oriented to an alternative fresh market seeking mildly pungent rocotos with high sweetness, visual versatility, and moderate fruit moisture content; (ii) Cluster 2, oriented to pungent fruits with elevated antioxidant capacity, in part associated with longer fruits; and (iii) Cluster 3, oriented to high fruit weight and high water content with a carotenoid emphasis, suited to processes where mass and red pigmentation are priorities (Figure 5). Geographically, the three clusters intermingle accessions from all zones (Arequipa, Ayacucho, Cajamarca, Huánuco, La Libertad, and Puno) without strict structure by provenance, reinforcing that group cohesion reflects morpho-functional profiles rather than origin (Supplementary Table S1).

### 3.5. Variability of Agro-Morphological Attributes by Zone of Origin

**Figure 6:**
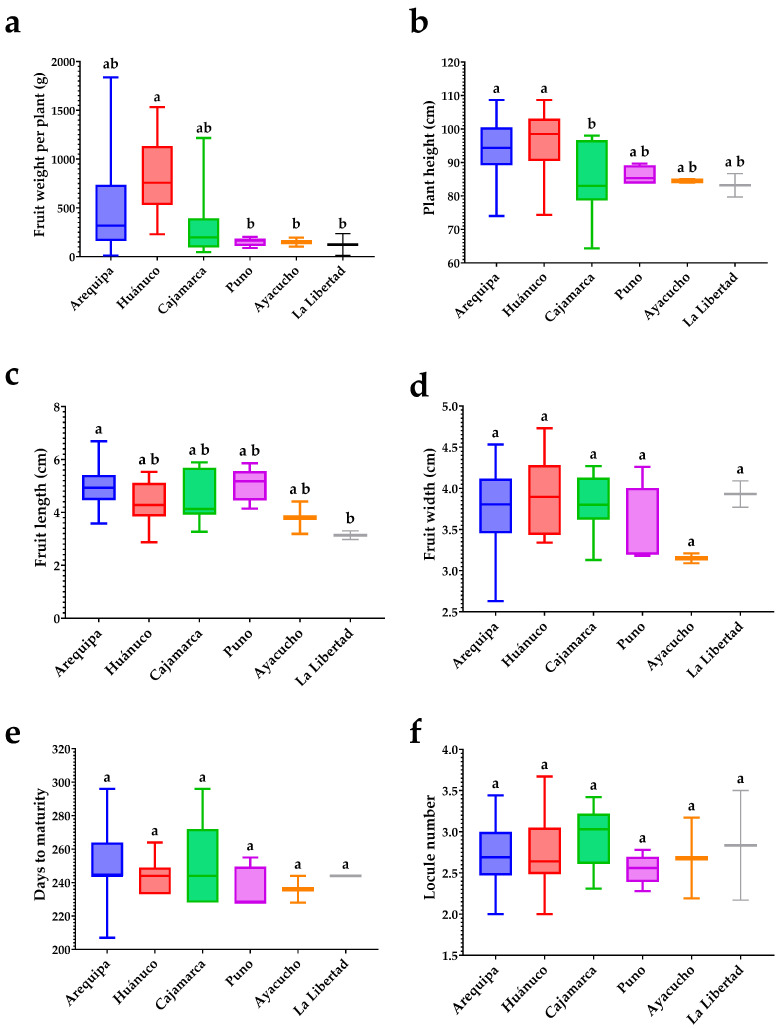
Comparison of means by zone of origin for agro-morphological variables in 78 rocoto accessions: (**a**) fruit weight per plant; (**b**) plant height; (**c**) fruit length; (**d**) fruit width; (**e**) days to maturity; and (**f**) number of locules. Boxplots show median (line), interquartile range (box), and data range (whiskers). Different letters indicate significant differences among zones (Dunn’s test, *p* < 0.05). For the cases of Ayacucho and La Libertad, due to the limited sample size, the reported values are intended only for comparative and reference purposes.

Figure 6 shows that fruit weight per plant, plant height, and fruit length differed among zones, whereas phenology (days to maturity) and locule number did not vary statistically across origins. Notably, only two accessions were evaluated from Ayacucho and two from La Libertad; therefore, the reduced variability observed in these zones likely reflects the very small sample size rather than true structural uniformity. In panel (a), fruit weight per plant discriminated zones: accessions from Arequipa and Huánuco concentrated the highest medians, and Huánuco exhibited a higher median fruit weight per plant than Cajamarca and Puno. A widespread is also evident for Arequipa, Huánuco, and Cajamarca, attributable to larger sample sizes and the presence of high-end outliers.

**Figure 7:**
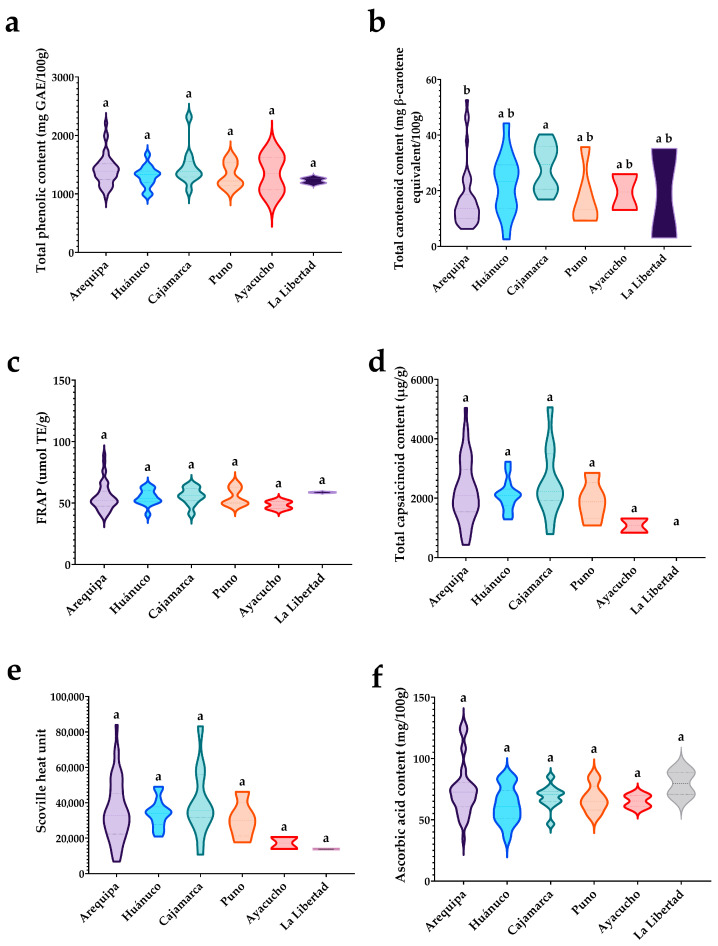
Distribution by zone of origin for functional variables in 78 rocoto accessions: (**a**) TPC (mg GAE/100 g), (**b**) total carotenoids (mg β-carotene equivalents/100 g), (**c**) FRAP (µmol TE/g), (**d**) total capsaicinoids (µg/g), (**e**) SHU, and (**f**) ascorbic acid (mg/100 g). Violin plots show kernel density; internal marks indicate median; different letters indicate significant differences among zones (Dunn’s test, *p* < 0.05). For the cases of Ayacucho and La Libertad, due to the limited sample size, the reported values are intended only for comparative and reference purposes.

In panel (b), the mean plant height of accessions from Arequipa and Huánuco was significantly greater than that of accessions from Cajamarca. In panel (c), accessions from Arequipa had a higher mean fruit length than those from La Libertad, whereas accessions from Huánuco, Cajamarca, Puno, and Ayacucho showed intermediate lengths. In panels (d–f), mean fruit width, days to maturity, and locule number did not differ statistically. This homogeneity suggests that fruit width, maturity timing, and locule number are stable across zones of origin, and that the geographic variation observed in the panel does not substantially alter these traits under the evaluated conditions. Overall, the figure indicates that geographic origin does not determine the average morphology or phenology of the accessions but is associated with differences in fruit weight per plant, with Huánuco concentrating accessions of higher yield potential. Figure 7 compares, by zone of origin, functional variables related to bioactive compounds, antioxidant activity, and pungency. The general pattern is clear: most functional traits did not differ among zones, and appreciable within-zone variability suggests broad scope for selection within each origin. For total phenolics (TPC; panel a), the five zones share the same letter, indicating no significant differences; nevertheless, violin widths are comparable with moderate tails, evidencing substantial internal dispersion in all provenances. Consistently, FRAP antioxidant capacity (panel c) also showed no zonal differences, with relatively compact distributions in Huánuco, Puno, and Cajamarca and somewhat more heterogeneity in Arequipa. The same was observed for ascorbic acid (ASC; panel f): statistically similar means with moderate spreads, except in Arequipa, which displayed considerable amplitude. Ayacucho, by contrast, showed the narrowest dispersion, attributable to the small sample size.

The only trait showing a zonal effect was total carotenoids (CAR; panel b). Accessions from Cajamarca displayed the highest mean carotenoid content relative to Arequipa, whereas Huánuco, Puno, and Ayacucho were intermediate and not distinct from either extreme. The violin shapes reinforce this conclusion: Cajamarca exhibits a distribution shifted toward higher values, while Arequipa concentrates density at the lower end. This separation suggests that, within the panel, carotenoid accumulation is the functional component that best discriminates geographically. For total capsaicinoids (TCS; panel d) and pungency (SHU; panel e), no differences were detected among zones; however, the violins reveal marked within-zone heterogeneity, especially in Arequipa and Cajamarca, with long upper tails indicating that very pungent accessions coexist with more moderate ones within the same provenance. As expected, TCS and SHU show parallel dispersion patterns, confirming that capsaicinoid variation accounts for most of the variation in Scoville units. Taken together, Figure 7 indicates that geographic origin does not consistently determine phenolics, FRAP antioxidant activity, capsaicinoids, pungency, or ascorbic acid; the only robust territorial gradient is observed for carotenoids. Operationally, this implies that (i) selection for phenolics, antioxidant activity, and pungency can be focused within each zone (the useful variation is intrazonal), and (ii) when the breeding or product goal is pigmentation and/or carotenoids, Cajamarca offers an average advantage, whereas Arequipa will require a finer search for standout accessions.

### 3.6. Promising Accessions

**Figure 8:**
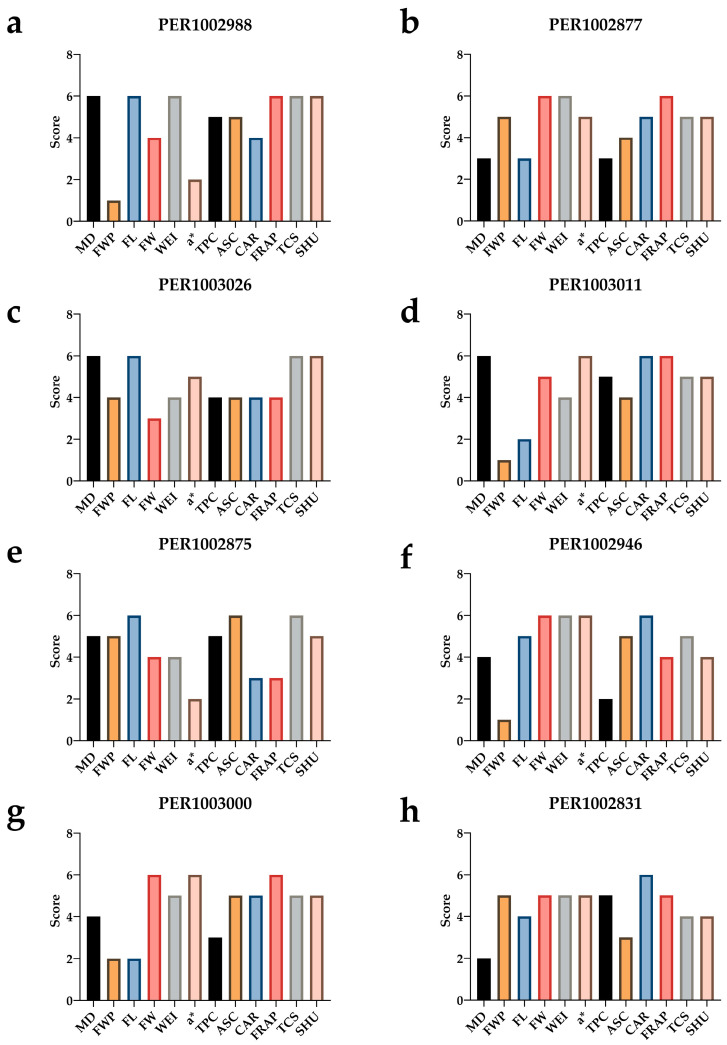
Multi-trait selection profiles of the top eight ranked rocoto genotypes: standardized scores (1–6) for agro-morphological, color, functional, and pungency variables; subpanels (**a**–**h**) correspond to 1st through 8th rank. For MD (days to maturity), scores are inverted (higher value = greater earliness).

**Figure 9:**
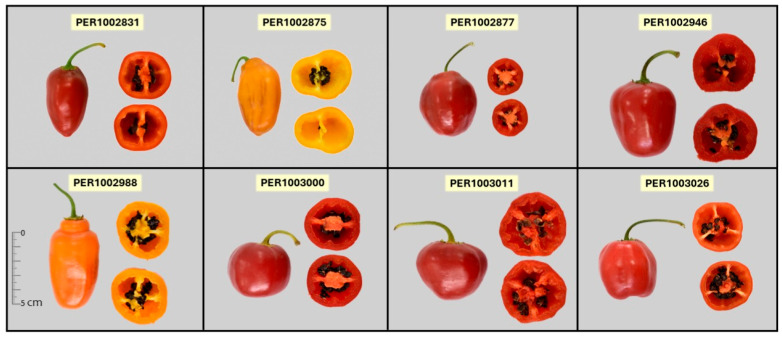
Promising accessions from INIA’s National Rocoto Collection (Peru) identified according to the multi-trait selection profiles of the top eight ranked genotypes.

Figure 8 synthesizes the multi-trait selection of the eight top-scoring accessions (Supplementary Table S2). The left-to-right order reflects the global ranking (a = first; h = eighth), and each bar summarizes relative performance in phenology, plant/fruit architecture and yield (days to maturity, fruit weight per plant, and fruit length, width, and individual weight), fresh-fruit CIELAB color (a*), functional quality (total phenolics, FRAP antioxidant activity, ascorbic acid, carotenoids, and total capsaicinoids), and pungency (SHU). The leading genotype (Figure 9), PER1002988, exhibits a very early profile, with long, wide, well-weighted fruits but low yield, a less intense red coloration, and outstanding levels of bioactive compounds, antioxidant activity, and pungency, an attractive candidate for a premium market that values elevated nutraceutical potential. Similarly, PER1002877 stands out for intense red color and an antioxidant bonus supported by phenolics; it maintains high carotenoids and pungency, with intermediate earliness and high fruit weight and yield, profiling it as a strong breeding candidate for a dual-purpose variety combining high yield with high bioactive content. PER1003026 is positioned as a spicy–antioxidant type, with good pungency and deep red color, plus good earliness, albeit with medium fruit weights and good per-plant yield. PER1003011 adds the virtue of very high earliness with intensely red fruits, though with lower yield and medium fruit weight, but noteworthy bioactive content. The remaining genotypes vary in specific attributes, yet all show solid nutraceutical potential, combining pungent fruits with high carotenoids and antioxidant activity. Collectively, the eight materials cover complementary niches, enabling the design of selection portfolios tailored to distinct end uses without sacrificing agronomic coherence or quality.

## 4. Discussion

The results obtained in this study constitute one of the most comprehensive morpho-functional profiles published to date for *Capsicum pubescens* (rocoto) germplasm. The remarkable intraspecific variability observed, from plant architecture and phenology to yield and fruit quality, aligns with previous reports indicating that rocoto is a little-studied yet highly variable domesticated *Capsicum* [38]. The broad range in fruit yield per plant (0.01–1.84 kg) with a coefficient of variation exceeding 90% reflects the presence of high-yielding accessions, consistent with Acunha et al. [16] who evaluated a Brazilian *Capsicum* collection (*C. annuum*, *C. baccatum*, *C. frutescens*) and identified genetic resources with agronomic and nutritional attributes. Notably, our rocoto accessions showed weak grouping by geographic origin (accessions from different Andean regions were intermingled among phenotypic clusters), suggesting that rocoto’s morpho-functional diversity is not strictly structured by provenance. This finding agrees with recent genomic studies [9], indicating substantial gene flow and limited population differentiation across the species’ range. In practical terms, desirable traits such as large fruit size or high phytochemical content are not confined to a single region, which is advantageous for breeding because diverse origins can contribute equally superior alleles.

With respect to agronomic variables, plant height and days to maturity showed low dispersion (PH, 9.76%; DM, 6.76%), indicating consistent, stable data, and they were positively correlated (0.23), mirroring observations by Moon et al. [39] in 513 accessions from two *Capsicum* species (*C. annuum* and *C. frutescens*). In that study, the coefficient for days to 50% maturity was 5.35% (low variability), whereas plant height had a coefficient of 24.51% (high variability), and both traits exhibited a significant positive correlation of 0.23, similar to our findings. The low variability observed for plant height in our *C. pubescens* panel indicates a narrower genetic breadth for this trait, potentially reflecting greater phenotypic uniformity within *C. pubescens* than the broader diversity documented for *C. frutescens* and *C. annuum*.

For fruit morphology, accessions exhibited moderate variability in length (2.87–6.69 cm), width (2.63–4.73 cm), and pericarp thickness (3.11–4.77 mm), with distributions near normality. Individual fruit weight and edible portion weight were positively correlated with dimensional variables, as is common in fleshy fruit species, while locule number was positively associated with width and negatively with length, replicating patterns reported in other *Capsicum* [24]. Soluble solids averaged 8.46 °Brix (range 6.60–11.40 °Brix), within typical values for moderately pungent chiles [16]. An inverse relationship was evident between fruit size and water content (fruit moisture content) versus soluble solids in our data: larger, juicier fruits tended to be less sweet, consistent with the well-known physiological dilution effect in horticultural crops [40]. This trade-off between yield and °Brix is well documented; for example, Lahbib et al. [41] reported that fruit agro-morphological and biochemical features were major contributors to overall phenotypic diversity in Tunisian peppers. Likewise Bakr et al., [42] described that tomato fruits from higher-yielding plants typically show lower soluble-solids concentrations due to dilution

Despite this antagonism, our principal component analysis suggests room for balance: several Cluster 3 rocoto accessions achieved above-average fruit weight without excessively compromising °Brix (i.e., only slightly below-average sweetness). Practically, this indicates that breeders can increase yield (fruit size/weight) while maintaining acceptable flavor, especially because fruit color and pungency in rocoto appear largely independent of sugars—an appealing feature for the market. Indeed, fruit color variables spanned a wide range, from yellow to red at full maturity, with substantial variation (CV up to 41.97% for the b* yellow–blue axis); however, these did not strongly correlate with sweetness. Such extensive color polymorphism is expected given rocoto’s range of pulp colors (yellow, orange, red) and aligns with Arredondo-Valdez et al. [23] in an unrelated crop (prickly pear), where morphological and colorimetric descriptors revealed high intraspecific diversity. These quantitative color data are valuable, as visual appeal (color, size, texture) is a key quality attribute for fresh markets.

In our panel, vitamin C (ascorbic acid) ranged from 34 to 128 mg/100 g dry weight, total phenolics from 945 to 2317 mg GAE/100 g, and total carotenoids from 2.5 to 52.6 mg β-carotene equivalents/100 g. Although the samples were not exposed to direct solar radiation but dried under screenhouse conditions, solar drying may negatively affect ascorbic acid content, mainly due to the effect of temperature. In this context, traditional solar drying has been associated with reductions of 83–86% in ascorbic acid content [43]. Likewise, Owusu-Kwarteng et al. [44] reported losses of 41.3% at drying temperatures of 55–69 °C and 43.3% at 69–77.5 °C in *Capsicum annuum*, indicating that increasing thermal conditions during solar drying reduces the retention of this vitamin. Even drying in a hot-air chamber at 60 °C can result in more severe degradation, with residual levels close to 10% [45]. Therefore, although vitamin C (ASC) contents may be affected by the drying process, the results are suitable for relative comparison among accessions, but do not reflect the absolute nutritional value of fresh food. Because the evaluation was conducted in a single environment, the functional data are interpreted primarily in terms of relative accession ranking. The values obtained in this study are comparable to those reported for *Capsicum* spp. elsewhere; for instance, the Brazilian pepper germplasm studied by Acunha et al. [16] exhibited similarly wide differences in total phenolics among accessions, and Wahyuni et al. [46] documented large disparities in metabolites (phenolics, flavonoids, vitamins) across 32 diverse pepper accessions. Our findings reinforce that rocoto, although a relatively under-domesticated Andean crop, harbors nutraceutical levels comparable to the widely studied *C. annuum* complex [47,48,49]. *Capsicum* fruits are broadly recognized as rich sources of bioactives, ascorbic acid, carotenoids, flavonoids, and capsaicinoids, with implications for human health [14,15]. Metabolite concentrations depend on genotype and environment, as noted by de Sá Mendes y Branco de Andrade Gonçalves [15]. In our study, genotypic effects predominated, with up to ninefold differences between the highest and lowest accessions for antioxidant activity (37.77 vs. 4.09 μmol TE/g, DPPH) and two- to fourfold differences for total phenolics and vitamin C (Table 1). This magnitude of functional variation in rocoto can be complemented with agro-morphological variables for improved selection in breeding and for establishing species-specific phytochemical baselines [8]. Rocoto also exhibits a distinctive capsaicinoid profile; dihydrocapsaicin (DHC) often rivals or exceeds capsaicin (CAP) in concentration, in contrast to most peppers where capsaicin predominates, as reported by Alghamdi et al. [50]. Our data corroborate this: mean DHC (871.90 μg/g) slightly exceeded CAP (802.50 μg/g), and DHC constituted just under half of total capsaicinoids on average. This agrees with other reports that *C. pubescens* has an unusual capsaicinoid signature with a higher DHC:CAP ratio compared with capsaicin-rich profiles (60–75%) typical of *C. chinense* or *C. annuum* [50,51,52]. In practice, this chemical profile could influence both perceived pungency and antioxidant properties, since structural differences among capsaicinoids affect pungency intensity and kinetics. Capsaicin and dihydrocapsaicin together account for >90% of capsaicinoids in hot peppers [18,53]. Kollmannsberger et al. [7] further found that rocotos possess capsaicinoid and volatile compositions distinct from other cultivated species. Absolute pungency levels in our collection (6814–83,961 SHU) fall within previously reported ranges for rocoto types [8]. Although some publications cite 130,000–162,000 SHU for certain varieties [54], such extremes likely apply to selected genotypes. Most of our accessions were moderately pungent (median 32,989 SHU), consistent with the notion that rocotos are typically hot but generally not as pungent as the hottest *C. chinense* cultivars [7,55]. Interestingly, van Zonneveld et al. [56] observed that local rocoto landraces with mild heat and high nutritional value (elevated vitamins and antioxidants) have attracted Peruvian entrepreneurs targeting health-conscious markets. This aligns with our Cluster 1, comprising accessions with low pungency, high soluble solids (sweetness), attractive bright yellow/orange colors, and low fruit moisture content, candidates for specialty fresh markets offering flavor and nutraceutical benefits without extreme heat. Conversely, our Cluster 2 phenotypic profile contained extremely pungent accessions with high antioxidant activity but lower sugar, ideal for dry spice products or hot sauces where high heat and antioxidant properties are desirable. A comparable grouping emerged in Lahbib et al. [41], for Tunisian peppers, where a subset showed elevated capsaicin and strong antioxidant activity. Together, these parallels underscore that *Capsicum* germplasm can be stratified into distinct phenotypic profiles aligned with different end-use targets, even within a single species such as *C. pubescens*.

Beyond capsaicinoids, carotenoid content in rocoto also warrants comparison [57]. Many red-fruited *C. annuum* cultivars (e.g., paprika, red chili) are known for high carotenoid content (capsanthin, β-carotene, lutein, etc.), exploited as natural colorants [58]. By contrast, *C. pubescens* has been reported as a relatively poor carotenoid source [59]. Our findings partly support this view: while a few red rocoto accessions showed considerable carotenoid levels (up to 52.59 mg β-carotene equivalents/100 g DW), the species average (19.62 mg/100 g) was not as high as some *C. annuum* varieties, suggesting that rocoto may have lower average total carotenoids, possibly due to genetic differences in pulp pigment biosynthesis [60]. Even so, we identified counterexamples grouped in Cluster 3 with high total carotenoids and deep red/orange coloration, indicating genetic potential within rocoto to select for enhanced carotenoids and narrow the gap with high-pigment species. Similarly, for phenolics, our rocoto mean (1.38 g GAE/100 g) was slightly higher than values reported for other hot chiles [61] and comparable to Vera-Guzmán et al. [51]. Lahbib et al. [41] found Tunisian peppers averaged 0.23–0.48 g GAE/100 g DW (translating to ~0.02–0.05 g/100 g FW depending on fruit moisture content) at full maturity. Thus, rocoto’s phenolic content falls comfortably within the range of its congeners. Notably, in our PCA, total phenolics contributed prominently to antioxidant activity (ABTS and FRAP), echoing results in other species [62]. For example, Lahbib et al. [41] observed close correlations between total phenolics and antioxidant activity across harvest stages. Collectively, these comparisons show that rocoto shares broad patterns of variation seen in other cultivated *Capsicum* while also exhibiting unique features (e.g., DHC-rich pungency profile). Rocoto therefore emerges as an interesting target for further research and as a potential genetic resource (e.g., tolerance to adverse environments or novel phytochemical profiles) in *Capsicum* breeding programs [63].

The identification of distinct phenotypic profiles in our rocoto germplasm parallels findings in other solanaceous crops and suggests breeding strategies that integrate yield and quality [2,64]. Three main phenotypic profiles were identified: a sweet, visually striking type (low pungency, high soluble solids, bright yellow–orange color); a functional high-pungency type (long fruits, very hot, with higher capsaicinoids and superior antioxidant activity); and a high-yield nutraceutical type (larger, heavier fruits with high fruit moisture content and exceptional carotenoid pigmentation). The third profile, exemplified by Cluster 3 accessions, represents an ideal phenotypic profile from both agronomic and nutraceutical perspectives: plants combined above-average fruit size and weight and overall yield with elevated levels of health-promoting compounds (especially carotenoids), doing so without extreme trade-offs (only slightly reduced sugars and moderate pungency). From a breeding standpoint, this is highly desirable; it indicates that wider, heavier fruits on high-yielding plants can also carry enhanced nutritional value (antioxidants, provitamin-A carotenoids), thereby advancing both agronomic improvement and nutraceutical quality within a single cultivar [65]. In essence, rocoto breeding could target combinations such as large fruit with lower sugar, high carotenoids, and high capsaicinoids to create dual- or triple-purpose cultivars, and our data suggests minimal physiological conflict when stacking these attributes. This scenario is analogous to tomato breeding, where modern varieties have been developed to produce large, high-quality fruits enriched in lycopene and other antioxidants [66], and to efforts in other solanaceous crops such as eggplant and potato, where breeders have successfully introgressed nutritionally beneficial traits (e.g., anthocyanin pigmentation, phenolic content) into high-yield backgrounds [67,68]. More broadly, the ideotype-breeding concept [69], which defines an optimal plant type and guides selection toward it, is applied here in a restricted, phenotypic sense. Our multivariate framework supports ideotype-oriented selection by identifying favorable trait combinations across agronomic and functional domains. By mapping these combinations to specific groups, we provide a practical selection model. For example, breeders could develop two divergent rocoto lines: one for the fresh market with sweet, mild, visually appealing fruits (Cluster 1 traits) and another for dry spices or hot sauces emphasizing maximal pungency and antioxidant activity (Cluster 2 traits). Our analysis also highlights potential to transcend traditional market segmentation: Cluster 3 effectively merges the fresh market’s emphasis on large, fleshy fruits with the processing market’s emphasis on bioactive content. Such “cross-over” superior profiles gain value as consumers increasingly seek products that are productive and flavorful yet also health-promoting. *Capsicum* researchers have emphasized that peppers offer a rich foundation for enhancing phytonutrients without sacrificing horticultural performance [47]. Our findings corroborate this in rocoto—an often overlooked species that can be repositioned as a nutraceutical crop given its concentrations of vitamins, phenolics, and carotenoids. We therefore recommend incorporating both agro-morphological and biochemical traits into selection indices for rocoto (and *Capsicum* more broadly) [41]. In this way, genetic gains in yield (e.g., heavier, wider fruits on productive plants) can be achieved while improving or at least maintaining levels of bioactive compounds that provide added value and health benefits. This dual-improvement strategy aligns with broader trends in crop breeding and food science that move beyond yield-centric goals to also encompass nutritional quality and functional properties [2]. Our study not only characterizes the rich diversity of Peruvian rocoto but also illustrates how this diversity can be leveraged to define ideal plant and fruit profiles. These profiles are promising for developing the next generation of rocoto cultivars that satisfy agronomists (high and robust yield) and consumers (enhanced nutraceutical content), thereby contributing to sustainable agriculture and public health [24]. Together, these results provide a preliminary phenotype-screening framework and species-specific quantitative baselines to guide superior phenotype-oriented breeding in *C. pubescens*. Accordingly, the within-accession sampling applied here is suitable for first-stage phenotypic screening. Given the open-pollinated nature of the germplasm, further validation through controlled selfing, progeny testing, and multi-environment trials will be required. Such trials could be conducted to assess genotype × environment interactions, determine the stability and adaptability of accessions under diverse conditions, and more accurately estimate phenotypic variability in both common-garden and multi-site contexts. These approaches would strengthen our understanding of accession performance and support more informed decisions in breeding and cultivation programs.

## 5. Conclusions

This study provides an integrated morpho-functional characterization of 78 *Capsicum pubescens* accessions from the Peruvian Andes, revealing high intraspecific variability in plant and fruit agro-morphological variables, physicochemical attributes, bioactive compounds, and antioxidant activity. Significant relationships were identified between structural fruit characteristics (e.g., weight and dimensions) and key functional variables such as total phenolics, carotenoid content, capsaicinoids, and antioxidant capacity. Hierarchical clustering distinguished three functional groups: Cluster 1 (light-yellow fruits with higher °Brix and lower fruit moisture content), Cluster 2 (long fruits with strong antioxidant profiles and higher pungency), and Cluster 3 (red, wide fruits with substantial carotenoid content), without strict geographic structure; by zone, Huánuco accessions stood out for yield per plant, and among functional traits only carotenoids showed geographic discrimination (Cajamarca > Arequipa; others intermediate). Multi-trait selection prioritized eight top-performing accessions; among them, PER1002988 combined earliness, large, heavy fruits, elevated bioactive profiles, and strong pungency (albeit lower yield and lower redness); PER1002877 integrated intense red color, high phenolics, carotenoids, and pungency with intermediate earliness and high yield, making it a dual-purpose candidate; PER1003026 defined a red, “spicy-antioxidant” phenotype with comparable earliness (medium fruit weight, good yield); and PER1003011 contributed very high earliness with red fruits and an attractive bioactive package. Overall, these results demonstrate that Peruvian rocoto germplasm enables the combination of desirable attributes—wide, heavy fruits on productive plants; high redness and carotenoids; capsaicinoids; and antioxidant capacity—without strong trade-offs across axes, providing a solid quantitative basis for future breeding efforts toward promising accessions with agronomic and nutraceutical potential, and for developing high-value cultivars suited to sustainable horticultural systems.

## Figures and Tables

**Table 1:** Descriptive statistics for 24 agro-morphological, physicochemical, bioactive compound, and antioxidant variables in 78 accessions of *Capsicum pubescens* from the National Rocoto Germplasm Collection (INIA, Peru).

Trait	Abbreviation	Unit	Mean	SD	CV	Min	Max	Median	Shapiro	Skewness	Kurtosis
Plant height	PH	cm	92.65	9.04	9.76	64.3	108.7	91.67	0.1601	−0.332	0.093
Days to maturity	MD	days	247.6	16.74	6.76	207	296	244	<0.0001	0.615	0.417
Fruit weight per plant	FWP	g	510.1	464.4	91	12.7	1838	295.5	<0.0001	1.169	0.526
Lightness (L*)	L	coordinate	46.63	9.36	20.1	33.7	59.11	53.05	<0.0001	−0.22	−1.855
Color coordinate (a*)	A	coordinate	26.24	6.58	25.1	14.3	37.52	25.83	0.0002	−0.019	−1.451
Color coordinate (b*)	B	coordinate	37.52	15.75	42	16.8	74.39	47.08	<0.0001	−0.071	−1.584
Chroma (C*)	C	coordinate	47.73	8.57	18	32.5	62.14	51.06	<0.0001	−0.108	−1.56
Hue angle (*h°*)	H	angle	51.69	18.09	35	26.5	73.45	62.06	<0.0001	−0.213	−1.882
Fruit individual weight	WEI	g	24.58	5.81	23.7	11.5	40.31	25.49	0.5153	0.037	−0.111
Fruit seed weight	FSW	g	0.70	0.20	29	0.28	1.17	0.68	0.2164	0.265	−0.668
Fruit edible section weight	EFW	g	23.88	5.72	24	11.1	39.31	24.72	0.5915	0.065	−0.118
Fruit length	FL	cm	4.70	0.85	18.1	2.87	6.69	4.64	0.8082	0.164	−0.347
Fruit width	FW	cm	3.76	0.45	11.9	2.63	4.73	3.79	0.2993	−0.267	−0.417
Fruit thickness	FT	mm	3.96	0.40	10.1	3.11	4.77	3.95	0.4081	−0.094	−0.65
Locule number	LN	value	2.74	0.38	14	2.00	3.67	2.69	0.3396	0.156	−0.639
Soluble Solids	BRIX	°Brix	8.46	0.94	11.1	6.60	11.4	8.33	0.1013	0.6	0.581
Fruit moisture content	HUM	%	89.41	1.13	1.26	86.9	93.44	89.45	0.0404	0.486	1.605
Total phenolic content ^1^	TPC	mg GAE/100 g DW	1382	252.6	18.3	945	2317	1360	<0.0001	1.285	2.916
Total carotenoids content ^1^	CAR	mg β-carotene eq/100 g DW	19.62	11.19	57	2.5	52.59	16.69	0.0001	0.947	0.295
Ascorbic acid content ^1^	ASC	mg/100 g DW	70.44	18.57	26.4	34.4	128	69.09	<0.0001	1.163	2.126
ABTS assay antioxidant activity ^1^	ABTS	umol TE/g DW	113.3	38.17	33.7	58.3	240	102.7	0.0017	0.799	0.433
DPPH assay antioxidant activity ^1^	DPPH	umol TE/g DW	16.82	7.02	41.7	4.09	37.77	15.21	0.0029	0.779	0.51
FRAP assay antioxidant activity ^1^	FRAP	umol TE/g DW	54.93	9.34	17	38.2	89.08	53.16	0.0006	1.116	2.296
Nordihydrocapsaicine ^1^	NHC	µg/g DW	486.4	253.1	52	93.3	1357	438.3	0.0033	0.892	0.91
Capsaicine ^1^	CAP	µg/g DW	802.5	419.4	52.3	113	2542	764.7	0.0007	1.177	2.996
Dihydrocapsaicine ^1^	DHC	µg/g DW	871.9	430.5	49.4	159	2391	804.8	0.0042	0.92	1.094
Total capsaicinoids content ^1^	TCS	µg/g DW	2161	989.3	45.8	428	5057	2071	0.0069	0.795	0.676
Scoville heat units	SHU	value	34,509	16,082	46.6	6814	83,961	32,989	0.0049	0.856	0.998

^1^: Results are expressed on a dry-weight basis (DW) for functional compounds, unless otherwise indicated. HUM corresponds to fresh fruit moisture content (FW). GAE: Gallic acid equivalent. β-carotene eq: β-carotene equivalent. TE: Trolox equivalent.

## Data Availability

The original contributions presented in this study are included in the article/Supplementary Materials. Further inquiries can be directed to the corresponding author.

## Supplementary Materials

The following supporting information can be downloaded at https://www.mdpi.com/article/10.3390/plants15020288/s1, Figure S1: Silhouette-based K selection; Annex S1: KMO and Bartlett’s tests and variance explained by principal component analyses (PCA 3A and PCA 3B); Table S1: List of clusters, origin zone, accessions and associated variables; Table S2: Complete list of scores; Table S3. Provenance data and mean values of 28 agro-morphological, physicochemical, colorimetric, bioactive, and antioxidant-capacity variables in 78 *Capsicum pubescens* Ruiz & Pav. accessions from the INIA National Rocoto Germplasm Collection (Peru); Table S4: Fertilization program applied in rocoto cultivation starting in May 2024; Table S5: Climatic data recorded at Santa Rita during the study period; Technical report S1: Quantification of Capsaicinoid Standards (Nordihydrocapsaicin, Capsaicin, and Dihydrocapsaicin).
