# Supplementary material for: Comprehensive Morpho-Functional Profiling of Peruvian Andean Capsicum pubescens Germplasm Reveals Promising Accessions with High Agronomic and Nutraceutical Value
Source: Plants (Basel). 2026 Jan 17;15(2):288. doi: 10.3390/plants15020288 (PMC12845375; doi:10.3390/plants15020288)
Supplement: Supplementary file 1 [file plants-15-00288-s001.zip › Supp Material_1.8_rev_MHG.pdf]

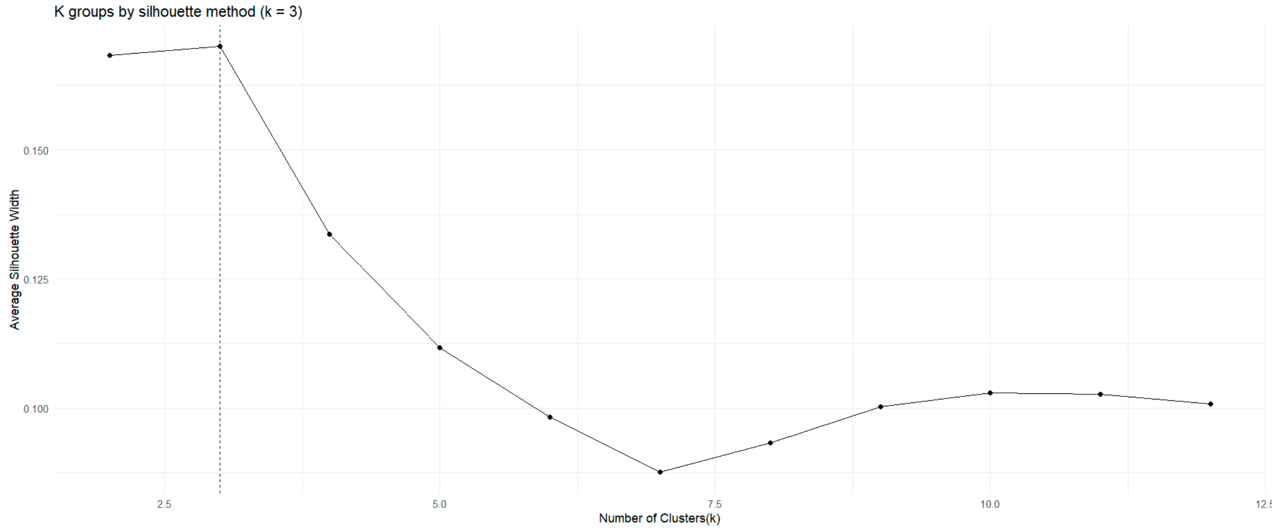

Figure S1. Silhouette-based K selection.

Annex S1. KMO and Bartlett's tests and variance explained by principal component analyses (PCA 3A and PCA 3B).

PCA 3A

Kaiser-Meyer-Olkin factor adequacy
Call: KMO(r = R)
Overall MSA = 0.68
MSA for each item =

PH	MD	FWP	FL	FW	FT	WEI	EFW	LN	FSW	L	A	B	C	H
0.51	0.64	0.39	0.58	0.85	0.51	0.54	0.53	0.65	0.30	0.80	0.74	0.90	0.82	0.76

> # Test de esfericidad de Bartlett

$chisq
[1] 2346.237

$p.value
[1] 0

$df
[1] 105

> print(pc_table[1:5,])

	PC	Eigenvalue	Var_percent	Cum_var_percent
1	PC1	5.088268	33.921786	33.92179
2	PC2	3.463047	23.086977	57.00876
3	PC3	1.390319	9.268791	66.27755

```
4 PC4   1.252716   8.351439      74.62899
5 PC5   1.110198   7.401321      82.03031
```

PCA 3B

```
Kaiser-Meyer-Olkin factor adequacy
Call: KMO(r = R)
Overall MSA =  0.71
MSA for each item =
   L    A    B    C    H BRIX  HUM  TPC  ASC  CAR DPPH ABTS FRAP  NHC  CAP  DHC  TCS  SHU
0.84 0.77 0.90 0.81 0.77 0.63 0.53 0.48 0.20 0.87 0.57 0.46 0.80 0.60 0.61 0.63 0.74 0.68
> Test de esfericidad de Bartlett
$chisq
[1] 5071.688

$p.value
[1] 0

$df
[1] 153

> print(pc_table[1:5, ])
   PC Eigenvalue Var_percent Cum_var_percent
1 PC1   5.487164   30.484243      30.48424
2 PC2   5.201778   28.898769      59.38301
3 PC3   1.881794   10.454411      69.83742
4 PC4   1.622931    9.016281      78.85370
5 PC5   1.092926    6.071808      84.92551
```

Table S1. List of clusters, origin zone, accessions and associated variables.

Cluster	n	Provenance zone	Accessions list (PER code)	Asociated variables
1	31	Arequipa	PER1002796, PER1002813, PER1002815, PER1002816, PER1002820, PER1002822, PER1002827, PER1002834, PER1002840, PER1002843, PER1002878, PER1002879, PER1002880, PER1002883, PER1002907, PER1002910, PER1002912 PER1002817, PER1002818, PER1002978, PER1002983, PER1002984	Fruit weight per plant, soluble solids, bright yellow-orange color
		Huánuco	PER1002860, PER1002863, PER1002870	
		Cajamarca	PER1003090	
		La Libertad	PER1003003	
		Puno	PER1003049, PER1003055	

		Ayacucho	PER1003067, PER1003073	
2	13	Arequipa	PER1002808, PER1002901, PER1002920, PER1002931, PER1002959, PER1002972, PER1002977, PER1002988, PER1002992, PER1002993, PER1002995	Fruit length, capsaicinoid content, pungency, and antioxidant activity
		Huánuco	PER1002875	
		Puno	PER1003048	
3	34	Arequipa	PER1002831, PER1002835, PER1002837, PER1002841, PER1002849, PER1002886, PER1002894, PER1002946, PER1002956, PER1002961, PER1002985	Fruit width, fruit weight, locule number, carotenoid conctent, and red color
		Huánuco	PER1002858, PER1002861, PER1002862, PER1002865, PER1002866, PER1002872, PER1002873, PER1002874, PER1002876, PER1002877	
		Cajamarca	PER1002996, PER1003000, PER1003004, PER1003006, PER1003008, PER1003011, PER1003016, PER1003021, PER1003026, PER1003028	
		La Libertad	PER1003001	
		Puno	PER1003059, PER1003063	

Table S2. Complete list of scores.

Accessions were ranked per trait across the full panel and assigned sextile scores (1 = lowest sextile; 6 = highest). The co mposite "SCORE" equals the sum of twelve variables scores (range 12–78). Abbreviations: MD, days to maturity; FWP, f ruit weight per plant; FL, fruit length; FW, fruit width; WEI, fruit weight; *a**, color CIELAB; TPC, total phenolic content; ASC, ascorbic acid content; CAR, total carotenoid content; FRAP, essay antioxidant activity; TCS, total capsaicinoids; SH U, Scoville heat unit.
The eight top-ranked accessions (based on FINAL SCORE) are PER1002988, PER1002877, PER1003026, PER1003011, PE R1002875, PER1002946, PER1003000, and PER1002831.

ACC	ZONE	MD	FWP	FL	FW	WEI	*a**	TPC	ASC	CAR	FRAP	TCS	SHU	FINAL SCORE
PER1002988	Arequipa	6	1	6	4	6	2	5	5	4	6	6	6	57
PER1002877	Huánuco	3	5	3	6	6	5	3	4	5	6	5	5	56
PER1003026	Cajamarca	6	4	6	3	4	5	4	4	4	4	6	6	56
PER1003011	Cajamarca	6	1	2	5	4	6	5	4	6	6	5	5	55
PER1002875	Huánuco	5	5	6	4	4	2	5	6	3	3	6	5	54
PER1002946	Arequipa	4	1	5	6	6	6	2	5	6	4	5	4	54
PER1003000	Cajamarca	4	2	2	6	5	6	3	5	5	6	5	5	54
PER1002831	Arequipa	2	5	4	5	5	5	5	3	6	5	4	4	53
PER1002873	Huánuco	5	4	5	5	5	4	3	1	5	6	5	5	53
PER1002959	Arequipa	4	6	6	3	6	1	5	4	1	5	6	6	53
PER1002995	Arequipa	4	2	5	5	6	2	6	3	1	6	6	6	52
PER1002894	Arequipa	1	2	3	6	6	5	3	6	6	3	5	5	51
PER1002901	Arequipa	4	6	3	6	6	1	5	5	2	6	4	3	51
PER1002956	Arequipa	2	1	4	5	5	5	4	3	5	6	5	6	51
PER1002992	Arequipa	2	1	6	3	6	1	5	6	3	6	6	6	51
PER1003008	Cajamarca	4	6	1	3	2	4	6	2	6	5	6	6	51
PER1003049	Puno	2	2	6	6	5	3	5	4	2	6	5	5	51
PER1002862	Huánuco	5	3	5	3	4	5	6	3	6	4	3	3	50

PER1002874	Huánuco	3	6	4	6	6	5	1	5	5	2	3	4	50
PER1002977	Arequipa	2	1	6	5	4	3	4	5	2	6	6	6	50
PER1002920	Arequipa	1	2	6	2	4	2	5	5	3	6	6	6	48
PER1002996	Cajamarca	1	2	2	5	5	5	4	4	4	4	6	6	48
PER1002835	Arequipa	2	3	4	6	6	4	6	3	4	5	2	2	47
PER1002861	Huánuco	3	4	5	3	5	5	4	1	6	3	4	4	47
PER1002931	Arequipa	4	1	4	6	6	2	6	2	2	4	5	5	47
PER1002961	Arequipa	6	4	3	4	4	5	2	6	5	4	2	2	47
PER1003016	Cajamarca	6	4	6	1	2	1	5	6	5	4	4	3	47
PER1002822	Arequipa	1	4	5	4	5	2	5	6	4	2	3	4	45
PER1002876	Huánuco	2	6	2	5	3	6	2	4	4	3	4	4	45
PER1002849	Arequipa	6	3	3	1	1	5	5	2	6	2	5	5	44
PER1002872	Huánuco	2	5	3	6	6	6	1	1	5	1	4	4	44
PER1002978	Arequipa	4	4	5	4	5	1	4	6	2	3	3	3	44
PER1003048	Puno	6	1	2	4	2	6	6	4	4	3	3	3	44
PER1002808	Arequipa	5	3	2	3	3	1	6	2	1	5	6	6	43
PER1002815	Arequipa	3	4	4	5	6	3	4	5	4	3	1	1	43
PER1002972	Arequipa	4	5	3	2	1	1	6	3	1	5	6	6	43
PER1002983	Arequipa	6	5	4	1	1	1	6	5	2	2	5	5	43
PER1003063	Puno	5	1	4	1	2	4	2	6	4	5	4	4	42
PER1002816	Arequipa	3	5	5	3	3	3	3	5	3	2	3	3	41
PER1002985	Arequipa	4	2	2	6	5	6	2	4	3	3	2	2	41
PER1002993	Arequipa	1	2	5	2	3	3	4	2	1	6	6	6	41
PER1003021	Cajamarca	4	3	4	4	5	4	1	3	6	1	3	3	41
PER1002858	Huánuco	5	6	2	2	2	5	2	2	5	5	2	2	40
PER1003067	Ayacucho	6	3	2	3	3	5	2	2	6	2	3	3	40
PER1002863	Huánuco	5	4	1	2	1	4	4	6	3	5	2	2	39
PER1002880	Arequipa	3	6	5	3	4	1	2	4	2	1	4	4	39
PER1002883	Arequipa	2	6	6	1	2	3	3	1	2	3	5	5	39
PER1003004	Cajamarca	1	2	6	2	4	6	3	3	6	4	1	1	39
PER1002817	Arequipa	3	6	3	3	3	2	3	3	2	4	3	3	38
PER1002827	Arequipa	1	4	1	1	1	4	2	6	4	4	5	5	38
PER1002860	Huánuco	5	5	2	2	2	4	3	1	1	5	4	4	38
PER1002865	Huánuco	3	4	1	6	5	3	1	1	3	3	4	4	38
PER1002866	Huánuco	2	6	1	2	1	6	5	2	5	4	2	2	38
PER1002878	Arequipa	3	5	2	6	5	1	3	5	1	1	3	3	38
PER1002912	Arequipa	1	5	6	5	4	4	1	1	2	1	4	4	38
PER1003001	La Libertad	4	3	1	3	2	6	2	4	6	5	1	1	38
PER1003006	Cajamarca	1	3	1	4	2	4	3	1	6	5	4	4	38
PER1002818	Arequipa	5	5	3	4	3	1	4	5	2	1	2	2	37
PER1003028	Cajamarca	6	1	2	4	3	6	2	3	4	2	2	2	37
PER1002837	Arequipa	2	4	2	2	2	4	3	1	5	2	4	5	36
PER1002879	Arequipa	3	6	3	2	1	3	1	6	3	1	3	3	35

PER1002886	Arequipa	3	6	4	4	3	4	1	4	1	1	2	2	35
PER1002813	Arequipa	1	3	3	4	4	4	4	2	4	3	1	1	34
PER1002841	Arequipa	5	3	3	1	1	6	4	2	5	2	1	1	34
PER1002910	Arequipa	2	2	4	5	4	2	2	3	2	4	2	2	34
PER1002796	Arequipa	5	3	1	2	2	3	6	1	3	3	2	2	33
PER1002870	Huánuco	3	6	1	4	3	2	3	2	1	2	3	3	33
PER1003003	La Libertad	4	1	1	5	3	3	2	6	1	5	1	1	33
PER1003090	Cajamarca	6	2	1	1	1	2	6	4	5	3	1	1	33
PER1002907	Arequipa	1	5	4	1	1	6	1	6	4	1	1	1	32
PER1003059	Puno	6	2	5	1	2	1	6	2	1	2	2	2	32
PER1002984	Arequipa	1	1	5	3	3	2	1	1	3	4	3	3	30
PER1002840	Arequipa	2	3	1	5	3	2	4	5	1	1	1	1	29
PER1002820	Arequipa	1	4	6	2	4	3	1	1	2	2	1	1	28
PER1003055	Puno	6	2	5	1	1	2	1	3	3	2	1	1	28
PER1002834	Arequipa	3	5	4	2	2	3	1	1	3	1	1	1	27
PER1002843	Arequipa	5	3	1	1	1	3	6	3	1	1	1	1	27
PER1003073	Ayacucho	5	1	3	1	1	1	1	2	3	1	2	2	23
