# Supplementary material for: Comprehensive Morpho-Functional Profiling of Peruvian Andean Capsicum pubescens Germplasm Reveals Promising Accessions with High Agronomic and Nutraceutical Value
Source: Plants (Basel). 2026 Jan 17;15(2):288. doi: 10.3390/plants15020288 (PMC12845375; doi:10.3390/plants15020288)
Supplement: Supplementary file 1 [file plants-15-00288-s001.zip › Technical Report S1_Quantification of Capsaicinoid Standards.pdf]

Multi Sample Summary

SAMPLE INFORMATION

Sample Name:	STD-3	Acquired By:	System
Sample Type:	Standard	Sample Set Name	18092025
Vial:	52	Acq. Method Set:	CAPSAICINOIDES_2
Injection #:	1	Processing Method	Capsaicinoides_26082025_
Injection Volume:	20.00 ul	Channel Name:	2475ChA ex280/em320
Run Time:	36.0 Minutes	Proc. Chnl. Descr.:	2475ChA ex280/em320

Date Acquired: 9/18/2025 5:01:29 PM PET
Date Processed: 10/7/2025 4:28:41 PM PET

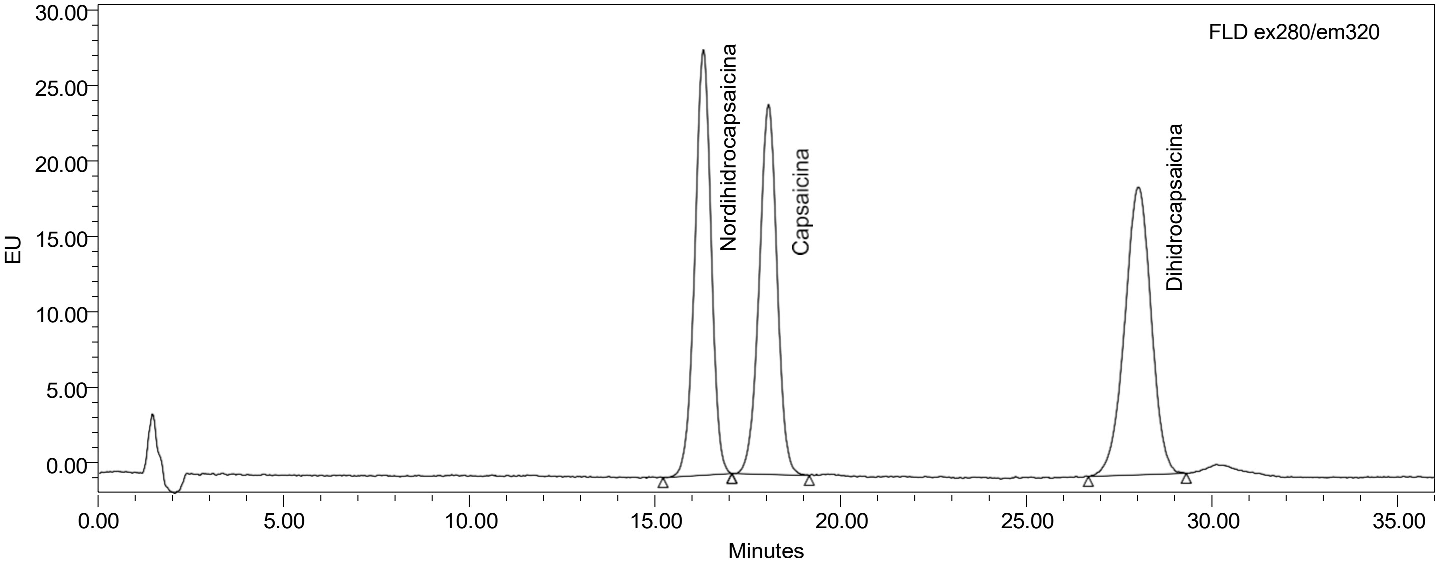

	Peak Name	RT	Area	% Area	Height	Amount	Units
1	Nordihidrocapsaicina	16.334	8596843	32.73	281913	0.600	mg/L
2	Capsaicina	18.087	8219953	31.29	245010	0.600	mg/L
3	Dihidrocapsaicina	28.044	9449320	35.98	190590	0.600	mg/L
